# Laboratory scale medicinal plants mediated green synthesis of biocompatible nanomaterials and their versatile biomedical applications

**DOI:** 10.1016/j.sjbs.2022.02.042

**Published:** 2022-03-01

**Authors:** Najlaa S. Al-Radadi

**Affiliations:** Department of Chemistry, Faculty of Science, Taibah University, P.O. Box 30002, Al-Madinah Al-Munawarah 14177, Saudi Arabia

**Keywords:** Green synthesis, Nanoparticles, Nanotechnology, Characterization, Applications

## Abstract

Nanoparticles and its green synthesis with plants have become an important field of nanoscience due it is great benefits provided to humanity through it and its cost effective, least harm to humans and the environment also, it offering a lot of application in biomedical research, diagnostics, and medicine as well as, drug manufacturing, improvement, or drug discovery. In this work, I focused on green synthesis nanoparticles with antioxidant involve in plants and the method for preparing them also the factors on which the extraction process depends on, spectroscopic techniques like UV–Visible, (TEM), (XRD), (IR), (EDX), (SEM), (HPLC), and zeta potential are use here.

## Highlights

• Different nanoparticles have been synthesized from different plants were summarized in this manuscript.
• This article emphasizes on to how nanoparticles can be easily synthesized using different plants extracts and what are their potential biomedical applications.
• Characterization was keenly comparative analyzed of different nanoparticles to find and suggest a new and sustainable way of biosynthesis of NPs.

## Graphical abstract

**Figure:**
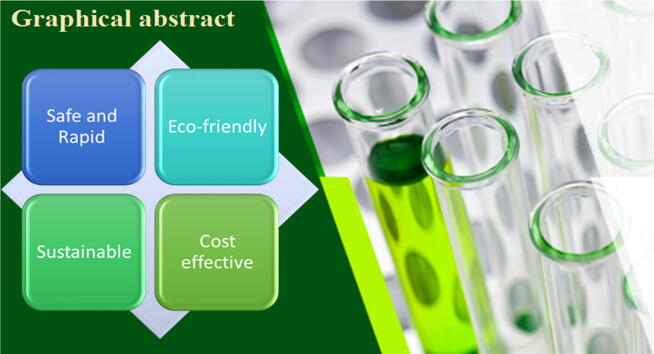

## 1 Introduction

### 1.1 Nano chemistry

**Fig. 1:**
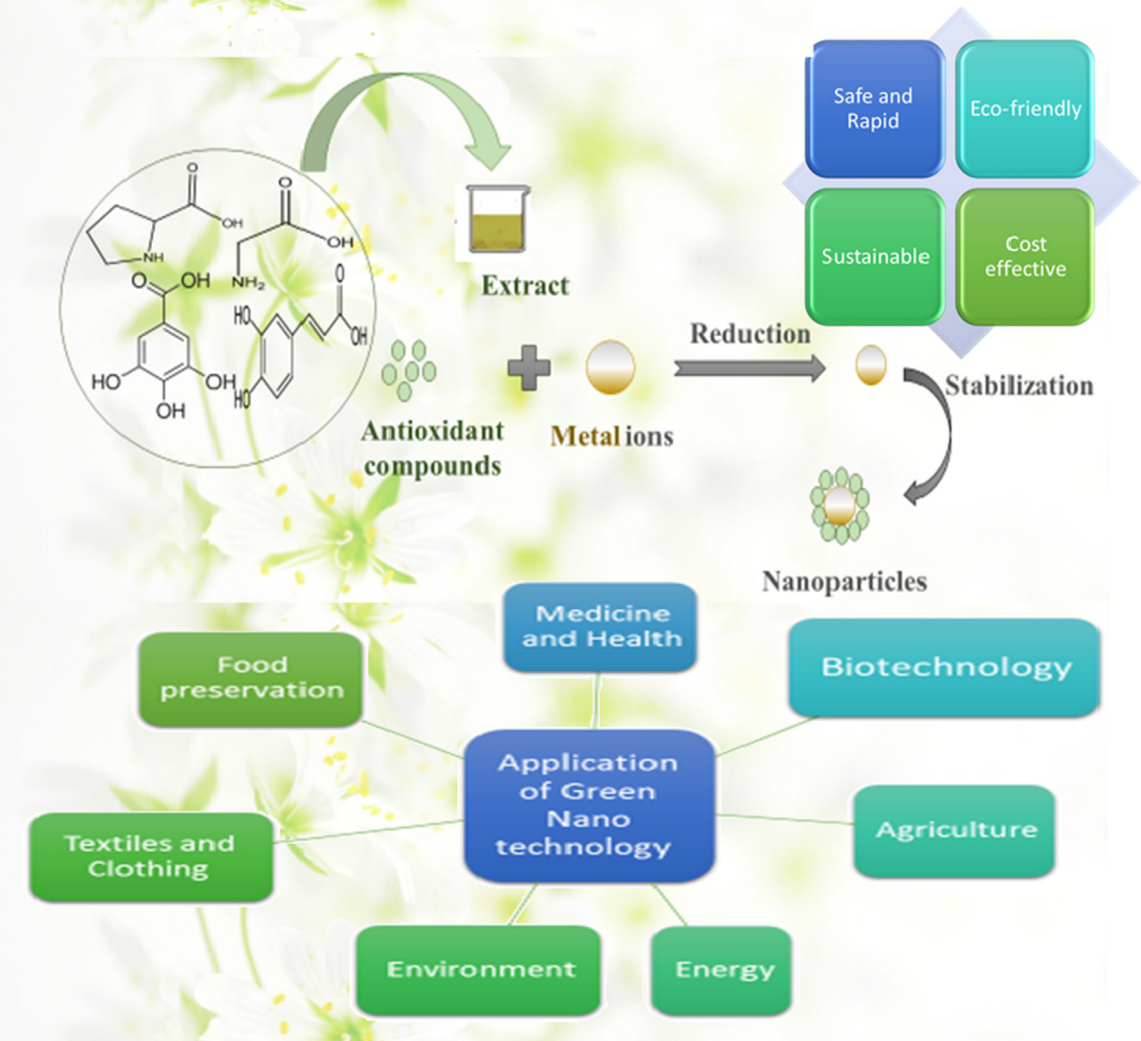
Scheme Plants Mediated Green Synthesis of Nanomaterials and their Versatile Applications.

Is there anything in science that doesn't include chemistry? Nano chemistry is playing a key role in nanoscience because of the bottom-up approach for the synthesis of polymers. The nano word means dwarf or small and its value is 10^-9^. The properties of a metal at nanoscale differs and show interesting properties. Aside from cosmetics, food, clothing, and household appliances, nanotechnology may be used to treat illnesses, distribute drugs, and create sustainable energy sources. Nanorods, nanowires, and nanotubes are intriguing nanotech products. The use of nanomaterials in nanomedicine is expanding. Although the choice of 10 nm appears arbitrary, its introduction helps to raise awareness of nano chemistry. This is also shown by the choice of (10) nm and the definition above. Because most harmful compounds have surface qualities, nanoparticles can remove them from water (Ozin & Cademartiri, 2009). Despite the early focus on nanoelectronics and future ideas, the first innovative and possibly practical nanotechnology to emerge from revolutionary nanoscience is he material synthesis. When addressing nanotechnology, it's easy to recognize the connection between green chemistry and the small scale. According to the definition given above, green chemistry uses harmless substances (Anastas & Eghbali, 2010). Plant extracts may be used to create nanometal, nanometal oxide, nanostructured polymers, dispersants, biodegradable polymers, and other products more eco- friendly. Most green chemistry materials are low-toxic and biocompatible because they use plants and biomaterials like proteins and lipids (Fig. 1).

### 1.2 Inorganic nano

NPs are microscopic entities having size in range of 1–100 nm. Nanopores, nanospheres, nanoclusters, hollow spheres yolk shells, nanorods, nanotubes, and core shells are all examples of attractive nanoscale morphologies (Sharma et al., 2015).

#### 1.2.1 MNPs fall into 4 divisions

Carbon-based, Organic-based, Inorganic-based, and Inorganic NPs. MNPs are metal and metal oxides, semiconducting materials with 2–10 nm diameter rare quantum dots**.** For the purposes of this definition, inorganic nanoparticles are particles that do not include carbon, metal, or metal oxide. There are many different metals used in the inorganic MNPs, including silver, gold, iron, aluminum, cadmium, cobalt, zinc, and copper etc. (Shah & Huseien, 2020). As the size of inorganic particles reaches the nanoscale scale, new physical features typically emerge. Inorganic nanomaterials must have their size, structure, and composition precisely controlled if they are to have desirable and frequently unique capabilities in sectors including biomedicine, catalysis, and sensing (Dahoumane et al., 2017). While organic nanoparticles have several uses in optics, electronics, magnetism, and catalysis, inorganic nanomaterials' properties may be modified by modifying their composition, structure, and form**.** Nanoparticles of inorganic nanoparticles such as silica give mechanical and thermal stability as well as unique capabilities based on chemical origin, shape, size, and crystallinity. Inorganic nanoparticles are commonly synthesized or produced due to their exceptional physicochemical properties. Nanomaterials of inorganic nature proved best among the nanoparticles. These nanomaterials have recently shown unique impacts in cancer imaging, diagnostics, and treatment, as well as molecular recognition devices and biomolecular transportation. Achieving stable, biocompatible inorganic nanoparticles with customized functional properties requires the development of homogenous, biocompatible inorganic nanoparticles. Metal and ligand complexes have functions like a reducing agent that reduces metal from its oxidation state to zero (M^+n^ to M^0^), and metals shrink in size. The other function of the metal–ligand compound is that it acts as an electron pair donor because the coordinate covalent bond between the metal and ligand is a Lewis acid that has empty orbitals (Fuente & Grazu, 2012). The production of nanoparticles using plant extract is superior to other approaches since it is straightforward, one step, cost-effective, ecologically friendly, and safe for clinical research to use. In order to improve efficacy and minimize adverse effects, these particles will be functionalized with antibodies or peptides for specific activity in selected regions of the body. In nanoparticles, the number of surface atoms reaches a considerable proportion of the total atoms, and surface energy plays an important influence in their characteristics (Wong & Schwaneberg, 2003). The absorption and interaction of (MNPs) with cells are not only controlled by their surface charge, but also by the associated ligands, therefore the coupling of ligands on the surface of (MNPs) might aid cellular binding (Khatami et al., 2016). It's also possible that surface ligands have a role in immune system activation. Metal nanoparticles made by plants have been shown to be more stable than those made by other organisms. Plants mediated synthesis of nanoparticles requires only one step rather than the multiple steps required by chemical methods (Berger, 2019). The electrons in the last shell of atom can be better explained by VSEPR and VBT theories (Kasote et al., 2015).

### 1.3 Antioxidants

Antioxidants are compounds that scavenge free radicals and plant extract has the power of doing it, therefore it is used for the NPs synthesis. Traditional medicines are now being revalued across the globe due to substantial study on many plant species and their medicinal properties. Studies have shown experimental evidence to support the theory that ROS and free radicals have a role in a wide range of illness (Scartezzini & Speroni, 2000).

#### 1.3.1 Why do all plants have antioxidant potential?

Plants have peroxisomes to invade free radicles such as reactive oxygen species (Río del et al., 2006). When these plant extract has combined with metal to form NPs have also a potent antioxidant activity (Khan et al., 2013). The antioxidant arsenal encompasses a diverse spectrum of antioxidant networks in cells, tissues, and bodily fluids, mirroring the diversity of prooxidants. Many benefits may be derived from photosynthesis of small particles by plants. Plant extracts may easily synthesize nanoparticles without the need for complex physical and chemical conditions (Phokha & Seraphin, 2015). Current research is focused on commercializing plants as sources of antioxidants (Reis et al., 2012). Phytochemicals have gained popularity as anti-aging dietary components due to their consistent antioxidant strength and cumulative antioxidant impact (Vayalil, 2012). Antioxidants have slow or stop the damage by attaching to metal ions and its oxides. Carotenoids and polyphenols are the most abundant antioxidants in fruits (Lim et al., 2007).

It is becoming more vital to do research on natural antioxidants in order to better understand the benefits of plant foods as well as how to improve the nutritional content of fatty ones by using natural antioxidants from fruits and vegetables. A growing number of dietitians, food makers, and consumers are taking an interest in natural antioxidants. Peels are a typical food waste that have received little attention due to their low economic worth (Okonogi et al., 2007).

#### 1.3.2 Fruits

Produce waste and leftovers from industrial processing of fruits and vegetables pose a severe concern since they have a negative influence on the environment and must be handled or used. Researchers have extracted from various peels a number of active compounds with properties such as those listed above as well as antioxidant and anti-proliferative properties (Parashar et al., 2014). Extracts of Purple apples from Yucatan, Mexico, were shown to be high in flavonoids and carotenoids, and high in antioxidant activity (ABTS) and DPPH) (Moo-huchin et al., 2015). The antioxidative activity of tamarind seed coat was attributable to the presence of acetate and benzoic acid as well as propyl esters, according to this study's findings. Avocado seeds are claimed to be rich in polyphenols and polymers such proanthocyanin (Soong & Barlow, 2004).

Many researchers found that orange have greater number of peroxidases than apple (Zia et al., 2011). Bioactive chemicals, such as phenolic compounds, are responsible for these positive benefits. In Passiflora spp. pulp, several phenolic compounds such as piceatannol and caffeic, coumaric and ferulic acids have been identified, and the *P. edulis* pulp is highly valued for its organoleptic qualities (Mariane Rotta et al., 2019).

There are many benefits of using orange peels and pulp in place of orange juice because oranges it is found worldwide and this can reduce pollution (Murador et., 2019). Nutritious orange peels may be utilized as medications or dietary supplements, whereas pulp is more antioxidant and antibacterial than peels (Arora & Kaur, 2013).

**Fig. 2:**
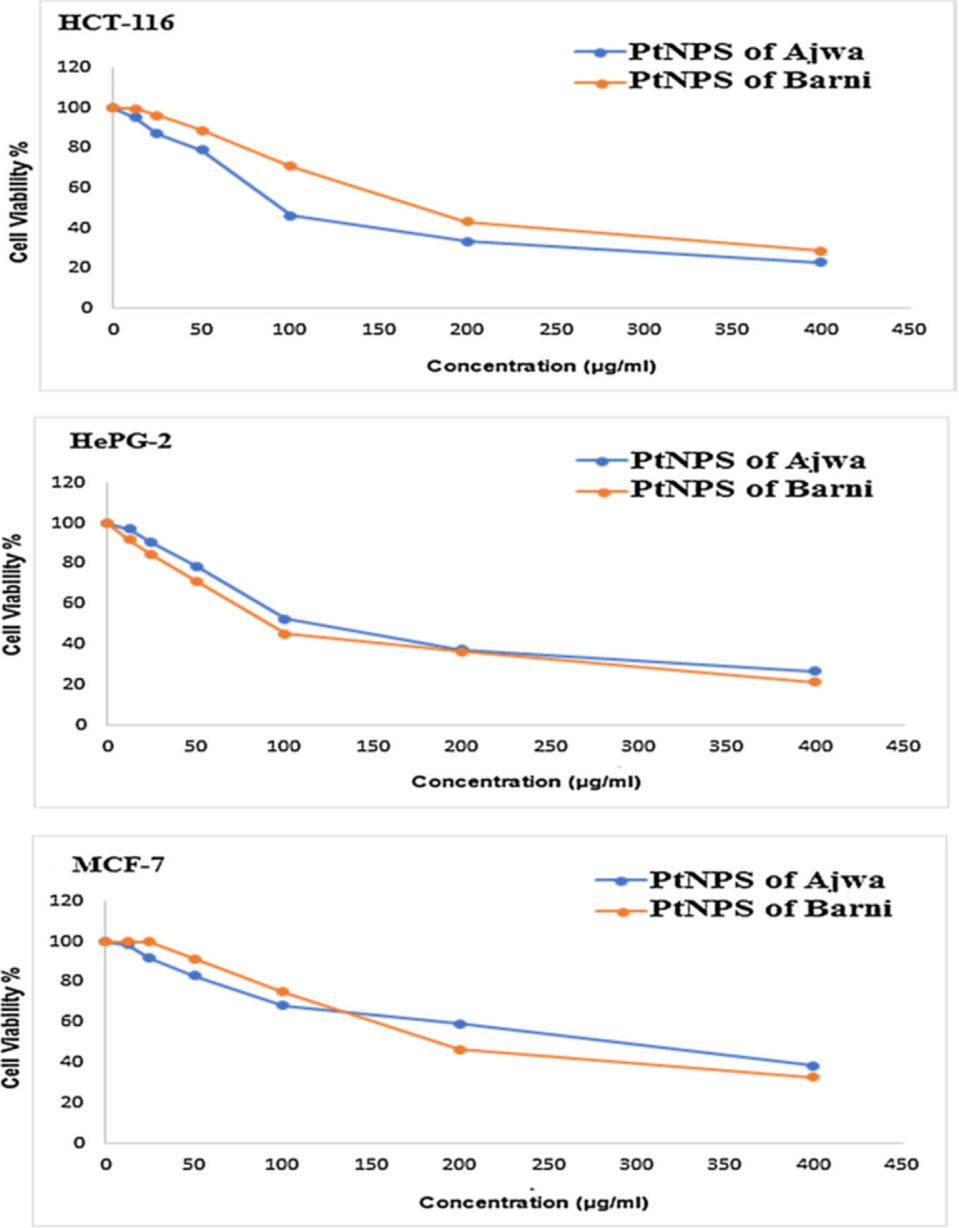
Inhibitory activity of synthesized PtNPs of Ajwa and PtNPs of Barni against human (HCT-116) cell line, (HepG-2) cell line and (MCF-7) cell line.

**Fig. 3:**
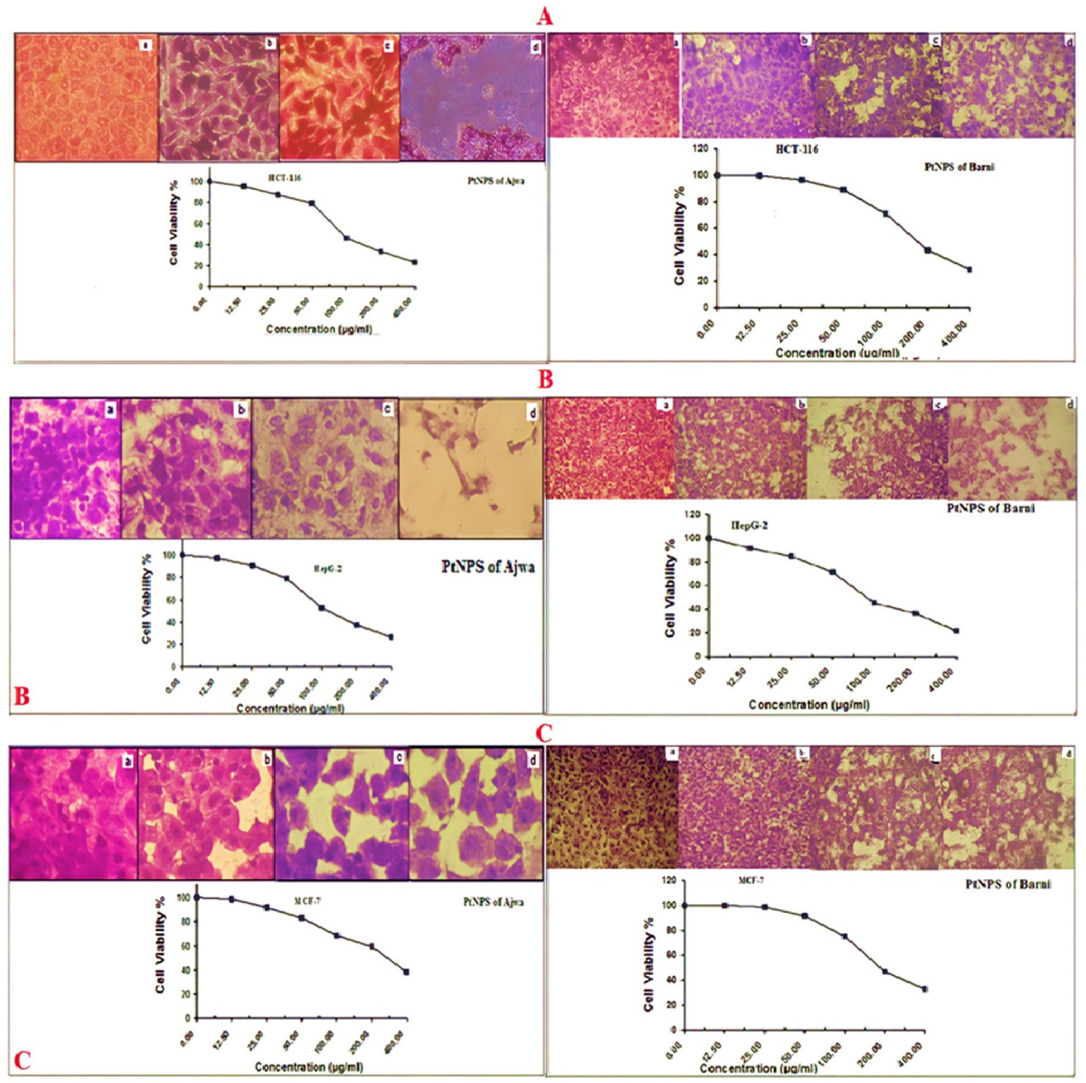
A) The photo image of coloncarcinoma cells (HCT-116) treatment by PtNPs of Ajwa and Barni, B) The photo image of Hepatocellularcarcinoma cells (HepG-2) treatment by PtNPs of Ajwa and Barni, C) The photo image of breastcarcinoma cells (MCF-7) treatment by PtNPs of Ajwa and Barni.

Avocado has a water content of roughly (80%) by weight. There is a lot of oil in the pulp, including phytosterols, carotenoids, aliphatic alcohols, tocopherols, and hydrocarbons. These phytochemicals from Avocado fruit help prevent heart disease and thrombosis, as well as cancer. Unsaturated fatty acids and phytosterols found in avocado oil make it a good source of anti-oxidants, anti-microbials, and anticancer agents in the food industry. Avocado oil can also be used in other ways besides as an edible oil. When it comes to nutritional value and therapeutic value, date plants from the AL-Maddinah AL-Munawarath regions like Anbara, Ajwi, Safawahi, Barni and green Ajwa are excellent choices. Dates have long been recognized to be high in antioxidants, antifungal, and antibacterial capabilities, among other benefits. In Fig. 2 there's a comparison between the inhibition of Ajwa and Barni. using Ajwa extract had a very promising results for colon carcinoma cells (HCT-116) it had about 76.82 % (HCT-116) > 73.24 % (HepG-2) > 61.53 % (MCF-7). Nonetheless, it gave different results using Barni's extract it had about 75.44 % (HepG-2) > 71.21 % (HCT-116) > 67.15 % (MCF-7) (Fig. 3) (Al-Radadi., 2019). Ajwa date extract has shown inhibition of MCF-7 cell line, this work investigates the biological potency of antioxidants from Barni and Ajwa date plants utilizing biosynthetic approaches. Ajwa dates include six vitamins and 23 important amino acids (Al-Radadi and Al-Youbi, 2018a, Al-Radadi and Al-Youbi, 2018b).

**Fig. 4:**
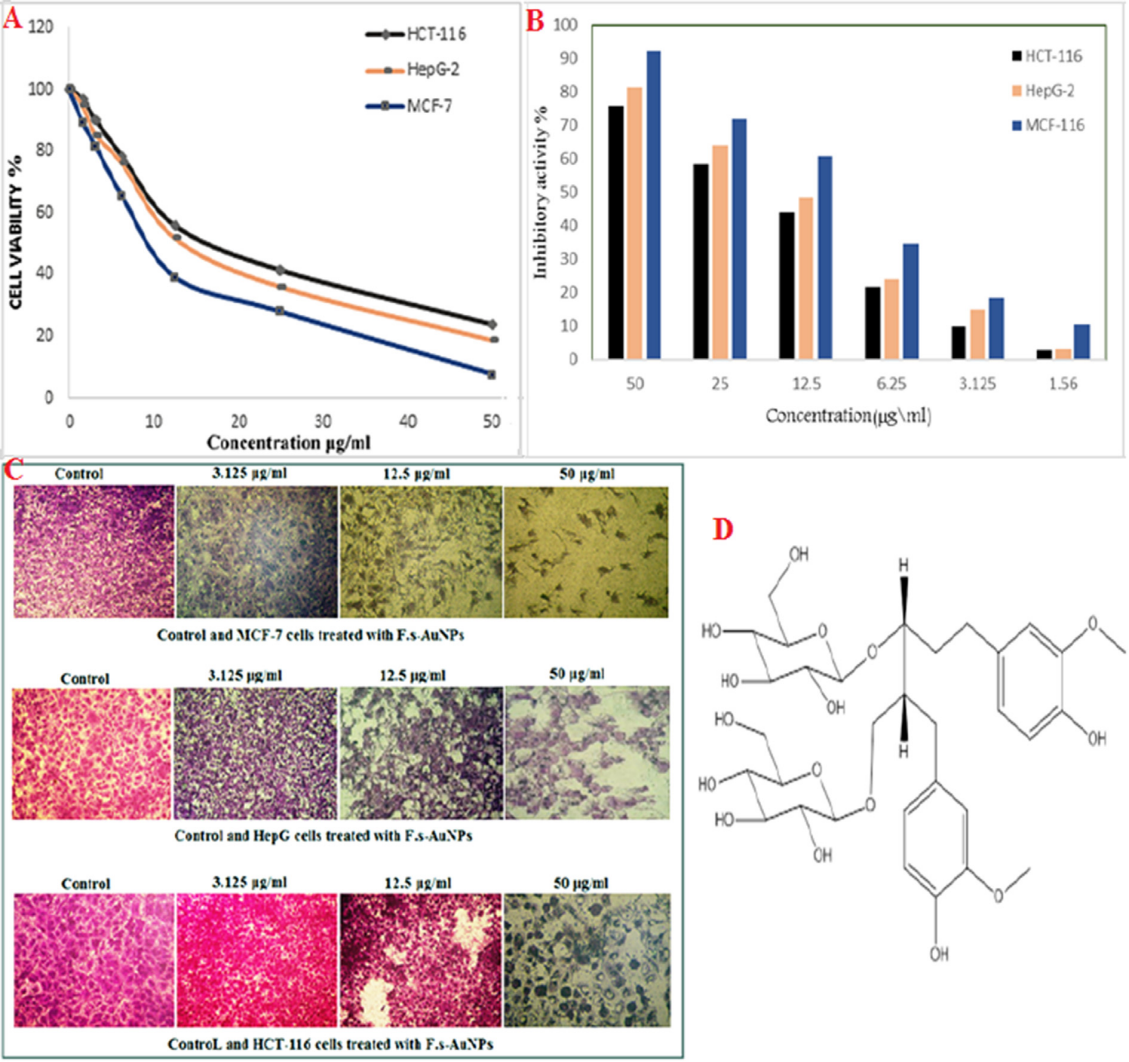
A) AuNPs percent cell viability B) AuNPs percent inhibition C) MCF-7, HePG-2 and HCT-116 cell lines inhibition by AuNPs, D) Chemical structure of secoisolariciresinoldiglucoside (SDG).

**Fig. 5:**
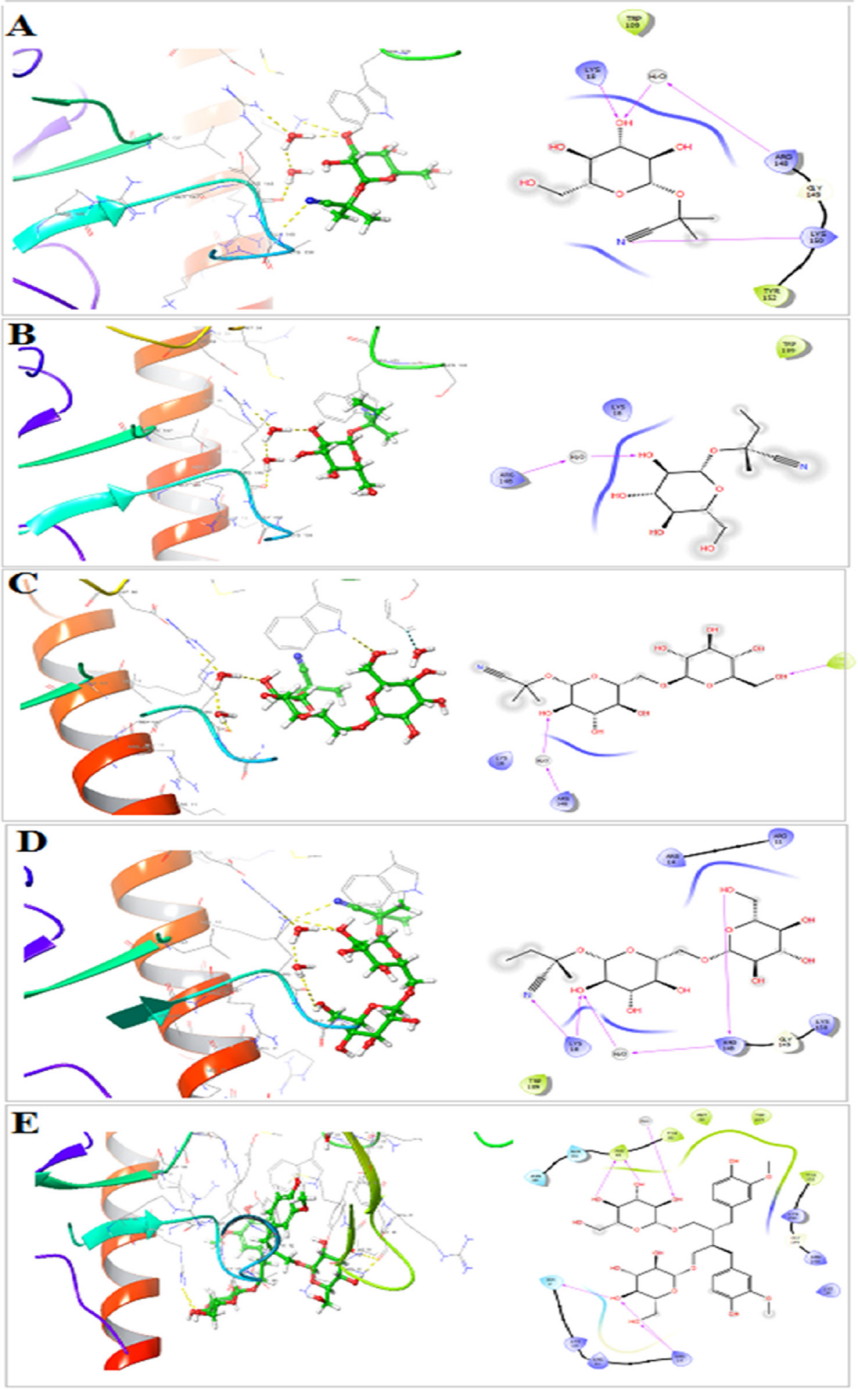
Molecular interactions of A) Linamarin, B) Lotaustralin, C) linustatin, D) neolinustatin, E) SDG to MCF-7 active sites.

The antioxidant activity of flaxseed@Au-NPs were equivalent to that of ABTS. Cancer cell lines including breast, hepatocellular, and colon cancer cell lines were significantly inhibited by mediated NPs (Fig. 4). SDG (secoisolariciresinoldi glucoside) has a strong affinity for MCF-7 in molecular docking. (Fig. 5) (Al-Radadi, 2021b).

#### 1.3.3 Herbs

Liquorice root, also known as Glycyrrhiza glabra L., is a popular Asian plant that's included in many dishes. In addition to its antioxidant, antimutagenic, and antidepressant properties, Licorice has almost 400 different chemical components (Al-Radadi, 2021a). Because of the pharmacological effects of these metabolic products, Licorice is broadly use as an anti-inflammatory. Licorice has been shown to have antiviral, cancer-fighting, antidiabetic, and expectorant qualities (Al-Radadi, 2022a, Al-Radadi, 2022b). An extract of the artichoke Cynara scolymus L., which has been shown to have antioxidative properties in the fight against free radicals and to be antibacterial, anti-HIV, and anti-inflammatory. Cynarin found in Cynara scolymus L. lowers (LDL) cholesterol while raising (HDL) cholesterol. Cynara scolymus L. has a high content of antioxidative substances, particularly polyphenol as the genus Calicotome has five different species, one of which is known as Calicotome villosa. Foruncle, cutaneous abscess and chilblain were treated with C. villosa, which was regarded an antitumoral agent and utilized in Sicilian folk medicine. The methanol extract and essential oil had antioxidant, cytotoxic and antibacterial action. Flavones, isoflavones, alkaloids, chrysin, and triterpenes are among the many compounds found in C. villosa (Al-Radadi, 2018). Known as anise or anise seed, *Pimpinella anisum* (commonly referred to as anise or aniseed) an animal herb. It is cultivated for its aromatic seeds, which are primarily used as spices and fragrances. The seeds of *P. anisum* have been used to treat cough, gynecologic, respiratory, neurologic, and digestive disorders. The antioxidant and antibacterial properties of anise extracts are good, indicating their potential utility in the treatment of infectious disorders and in the battle against free radicals (Zayed et al., 2020). The findings also show that ethanolic extracts have higher antioxidant and antibacterial activity than aqueous extracts, and this study sheds light on the antioxidant and antimicrobial properties of anise (Amer & Aly, 2019). Thyme (Lamiaceae) is used medicinally for its expectorant, antitussive, antibroncholytic, antispasmodic, anthelmintic, carminative, and diuretic properties. As well as therapeutic herbs, Thyme Thymus species have strong antioxidant activity and phenolic component concentration. Phenolic and flavonoids in thyme have anti-inflammatory properties (Wisam et al., 2018).

*Linaceae* is a commercially significant oil seed crop, with the seeds typically comprising (40%) of oil. Flaxseed is beneficial dietary item for human nutrition in addition to its traditional and has many biological applications (Al-Radadi, 2021b). Flaxseed protein included amino acids (Young et al., 2014). Saffron is highly prized for its bioactive properties, making it one of the costliest spices and medicinal plants (Ghanbari et al., 2019). Saffron, contain the compounds crocin, picrocrocin, and safranin, which are all degradation products of the carotenoid pigments. Picrocrocin gives saffron its bitter taste, while safranin gives it an aromatic quality. Crocus sativus is the only plant that produces significant amounts of these compounds. Considering that saffron petal is discarded in large quantities every year and contains substantial phenolic compounds with strong antioxidant strength, it's possible that phenolic compounds isolated from this solid waste might be employed as natural antioxidants (Amir et al., 2012).

#### 1.3.4 Vegetables

Cucumis sativus (cucumber) It has been used traditionally for headaches as a diuretic, nutritive, and demulcent, and as an emetic in acute indigestion in children (Nasrin et al., 2015). As a recognized provider of key nutrients such as carotene, a precursor to vitamin A, ascorbic acid, and various kinds of minerals, spinach is a widely produced worldwide dietary crop (Ko et al., 2014; Hatamjafari & Tazarv, 2013). Spinach have 10 different vitamins and vitamin C (Wong et al., 2018). Okra is a member of this family as well. The fruit mucilage is also used as an antioxidant and antiulcerogenic. Polysaccharide from Abelmoschus esculentus has hepatoprotective, antidiabetic, antiulcer, and anticancer properties (Ahmed & Kumar, 2016). The okra plant's vegetable and roots are rich in mucilage, which is commonly used as a food additive against gastric (Adetuyi & Ibrahim, 2014). The seed powder has also been used to purify water instead of aluminium salts. Okra seed is rich in vitamins and amino acids. Coriander is an annual herbaceous plant whose essential oil and different derivatives have been proven to contain antibacterial, antidiabetic, anticancerous and antioxidant properties, as well as antimutagenic and free radical scavenging activity (Kpodo et al., 2017). According to previous research, this plant has many therapeutic qualities, including anti-diabetic, antioxidant, hypocholesterolemic, antihelmintic, antibacterial and anxiolytic capabilities. For example, catechin, apigenin and petroselinic acid were identified in the C. sativum aerial portions, whereas p-coumaric acid, geranyl acetate and linalool were in the fruit (Fatima & Ahmad, 2005). Linoleic acid, oleic acid, and palmitic acid, are used in medicine as a carminative and diuretic, as well as in the production of numerous domestic medications to treat bed colds, seasonal fever, nausea, and stomach illnesses (Tang et al., 2013). Known as the common onion, Allium cepa is a widely-used flavorful vegetable across the globe. In addition to its antibacterial and anti-spasmodic qualities, onions include polyphenols, anthocyanins, flavonoids, quercetin, kaempferol, and their glycosides. Onions Allium cepa L. has distinctive fragrances and flavors that have made it a valuable culinary additive (Prakash et al., 2007). Recently, research has shown that onions have a number of biological qualities, such as antibacterial, antimutagenic, and antioxidant activities, which are commonly employed in food processing. Onion oil's organosulfur-containing constituents are medicinally relevant (Ye et al., 2013). Onion dry peel has powerful antioxidants and antidepressants (Singh et al., 2009).

Daucus carota L. is an Apiaceae root plant high in anthocyanins, carotenoid-carotene, vitamin A, B, and C. The hot water extract of the leaf is given orally during parturition as a uterine stimulant and has abortifacient potentials. The Apiaceae plants contain antioxidant and anti-inflammatory qualities and can be used to treat infections (Ayeni et al., 2018).

Parsley's major flavonoids are luteolin and its glycosides, which are abundant in the leaves. The flavonoid is anti-inflammatory, antioxidant, and anticancer (Papuc et al., 2016) Chemically, it has been discovered that parsley contains ascorbic acid, carotenoids, flavonoids, coumarins, apiole, and many other types of terpenoic chemicals, such as propanoids and phathalides (Mahmood et al., 2014). *C. pepo* is available throughout India and consumed around the world as a vegetable and have a wide range of flavonoids to be used as a herbal medicine (Labidi et al., 2021). In folk medicine, chard is utilized as an antidiabetic medication since it is a common plant in Turkey (Bolkent et al., 2000). Vegetable species of Beta vulgaris L. known as cicla According to some research, Chenopodiaceae may have hypoglycemic effects, and species of Beta vulgaris L. are often used as a folk cure for liver and kidney disorders, immune system activation, and a cancer treatment diet. Chard contains phospholipids, glycolipids, polysaccharides, ascorbic acid, and folic acid, as well as phospholipids, glycolipids, polysaccharides, and ascorbic acid (Sacan & Yanadag, 2010).

### 1.4 Antioxidant activity

During study on possible antioxidant function, researchers discovered 100 pure compounds (ROS) that start with oxygen and are spontaneously generated by enzymes in the cytoplasm (Lee et al., 2015).

**Fig. 6:**
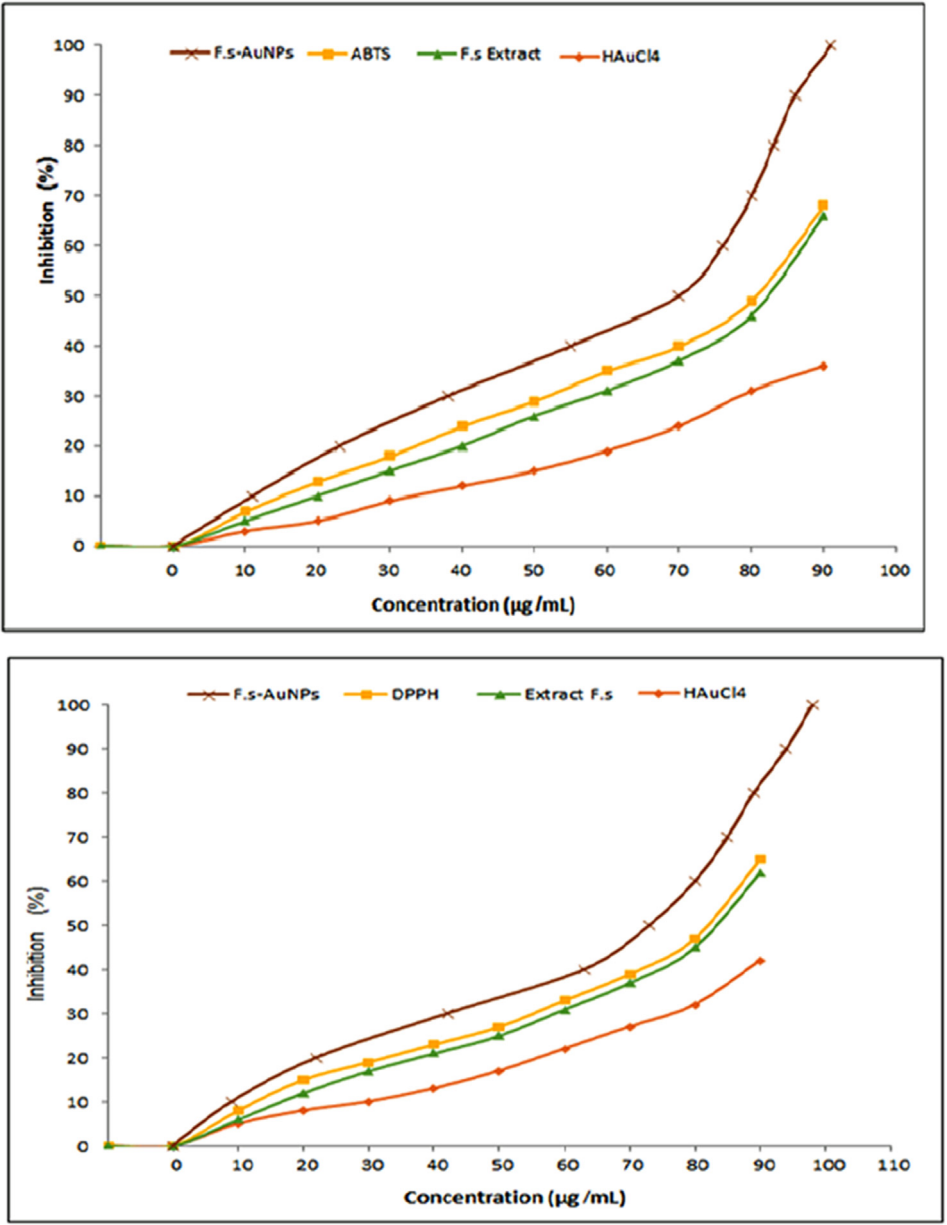
Antioxidant ABTS and DPPH Activity of AuNPs and Flaxseed Extract.

Several research on oriental medicinal herbs (OMH) have been undertaken to understand more about antioxidant activity that removes (ROS). (ABTS) and (DPPH) are two chemical scavenging strategies (Sricharoen et al., 2015). The most widely used spectrophotometric antioxidant tests are 2,2′-azino-bis-3-ethylbenzthiazoline-6-sulphonic acid (ABTS) and 1,1, diphenyl-2-picrylhydrazyl (DPPH) (Floegel et al., 2011). A spectrophotometer measures an organism's ability to scavenge or reduce a synthetic-colored radical or redox active chemical using an appropriate standard. Licorice root had high quantities of phenolics and ascorbic acid, which helped scavenge (ABTS) and (DPPH) radicals by supplying electrons and hydrogen (Al-Radadi, 2021a). The antioxidant activity of flaxseed extract (HAuCl4.3H2O) and (Fs-AuNPs) was assessed using (DPPH) and (ABTS) assays. The findings of the (DPPH) radical scavenging activity testing is presented in Fig. 6 (Al-Radadi, 2021b).

### 1.5 Fibrinolytic potential of activity MNPs

**Fig. 7:**
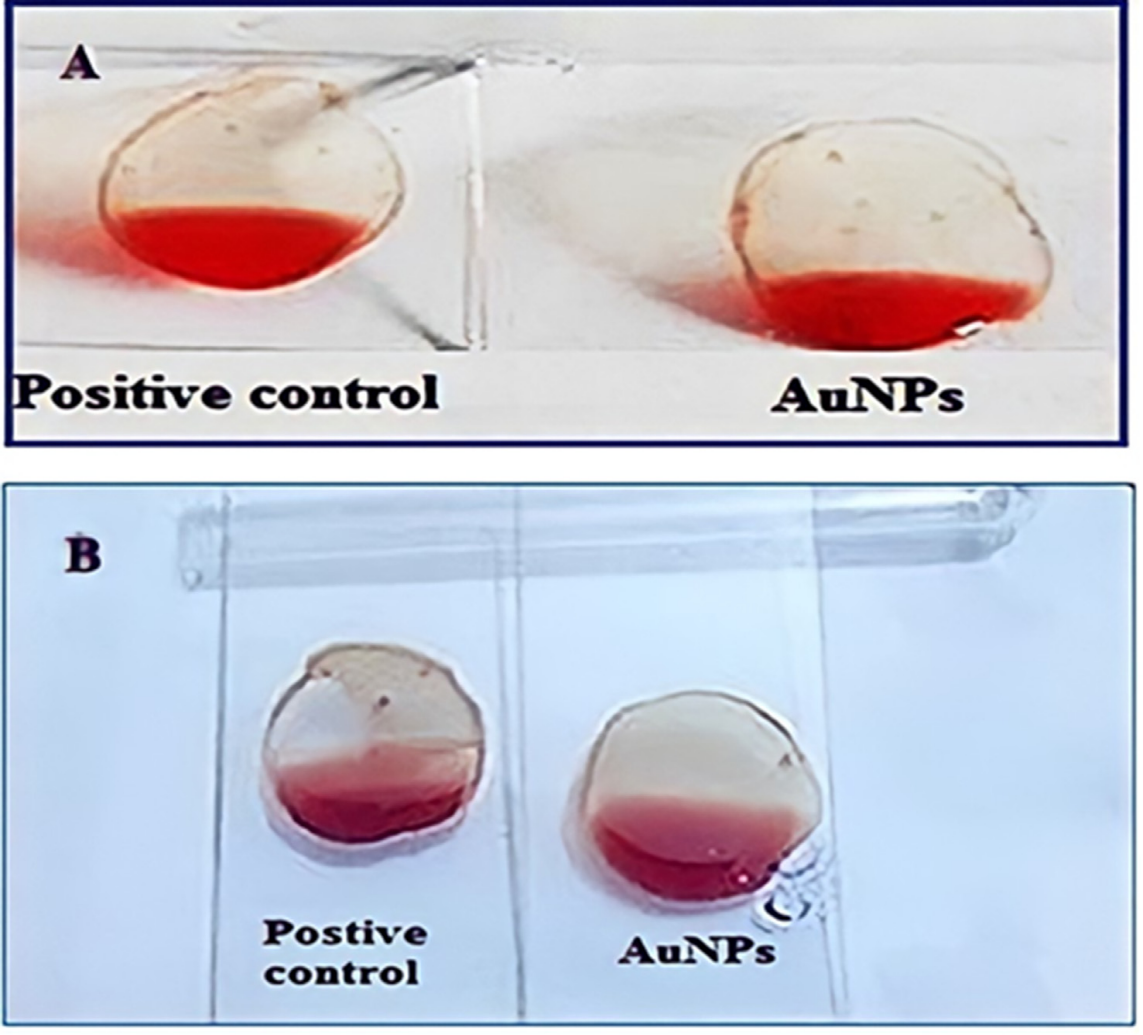
AuNPs Fibrinolytic Activity of A) Licorice root and B) Flaxseed extract.

Using an EDTA-based anticoagulant as a positive control, we tested the fibrinolytic efficacy of a fenugreek extract infused with gold nanoparticles. on a glass slide used as a positive control in the experiment. An extract of fenugreek containing gold nanoparticles was compared to an anticoagulant, EDTA, on a glass slide used as a positive control in the experiment. Licorice root and flaxseed extracts, as shown in Fig. 7**A and B**, had outstanding fibrinolytic action when mediated by AuNPs, supporting the anticoagulant function of human blood in this experiment. Microscopic analysis of the positive control revealed a substantial number of blood clots, even though RBCs were broadly distributed. (Al-Radadi, 2021a, Al-Radadi, 2021b).

### 1.6 Nanomaterial

The phrase “nanomaterial” refers to a material having a nanoscale external dimension, internal structure, or surface structure (Matteis & Rizzello, 2020). Using nanomaterials, we are able to study and create ultra-fine structures. To put it another way, we may consider both bottom-up and top-down ways to synthesizing nanomaterials. That is, we can either put together atoms or break down bulk solids into ever smaller fragments until they are made up of just a few atoms. Recently, biological nanoparticle synthesis utilizing plant extract. The compounds contained in plant extracts may also function as capping agents for nanoparticles during production, stabilizing them while decreasing their size (Shah et al., 2015). Green synthesis, which uses fewer reducing agents, surfactants, and stabilizers while generating fewer harmful derivative chemicals, has lately gained popularity. Preparation of nanoparticles utilizing sustainable synthetic activity reduces or eliminates the usage of an green chemistry techniques to synthesize nanoparticles offers various benefits over traditional approaches, such as being safe to handle, not polluting the environment, and reasonably priced Using green synthesis methods also has many additional benefits, including firstly the dual use of the active natural component, such as extract of plants (El Shafey, 2020). Second, the process of reducing metal ions takes just a brief time, and the final product is very stable because of the extract's inclusion of stabilizing chemicals (Mondal et al., 2020). Nanomaterials for energy conversion, storage, water purification, and building have all been explored as possible areas of application of green nanotechnology advancements, Other sustainable nanotechnology uses have also been researched (Ali & Ahmed, 2020). The use of manufactured nanoparticles in medical and environmental remediation has been a great success. A recent textile research found that (Ag-NPs) may have cytoprotective effect against HIV-1-infected cells. Ag-NPs may prevent HIV replication in Hut/CCR5 cells (Roy et al., 2013). These novel antibacterial have changed medicine (Herlekar et al., 2014). The dye removal effectiveness of (CuO-NPs) in actual water samples was found to be similar to that in ultrapure lab water (Chauhan et al., 2019). This could be because of the presence of various common cations, when it comes to environmental pollution abatement, iron nanomaterials play a critical role. This includes reducing organic dye degradation, removing chlorinated organic pollutants, and even removing heavy metals like arsenic. Other applications for iron nanomaterials include wastewater treatment, antibacterial activity, and plant mediated dye degradation (Saif et al., 2016).

### 1.7 Noble metal

Noble metallic nanoparticles are particles made from a noble metal that has size with in 100 nm. Catalyzed forms of the metals pose severe health risks to humans and the environment, as well as following cyclic paths to contaminate natural resources such as water or soil or even food (Amin et al., 2021). The majority of the transition metals have been found to pose multiple health risks even at trace concentrations. Noble metals have become a part of our everyday lives as a result of nature (Pradeep and Anshup, 2009). The synthesis of noble metals (MNPs) has resulted from the development of several methodologies (Doria et al., 2012). High surface to volume ratio, wide optical properties, are some of the distinctive features of noble metal (MNPs), and these features offer promise in the clinical area for cancer therapies (Conde et al., 2012). Capping agents are a common method for creating magnetic noble metal nanoparticles (Kowlgi et al., 2012). Compared to other materials, the most widely used metals are Au, Ag, and Pd. These metals have had a major impact on the disciplines of biology and medicine in the contemporary age (Yaqoob et al., 2020). A simple and effective green nano chemical process has been used for nanoparticles synthesis (Sanchez-mendieta, & Vilchis-nestor, 2012).

## 2 Experimental

### 2.1 Colors

**Fig. 8:**
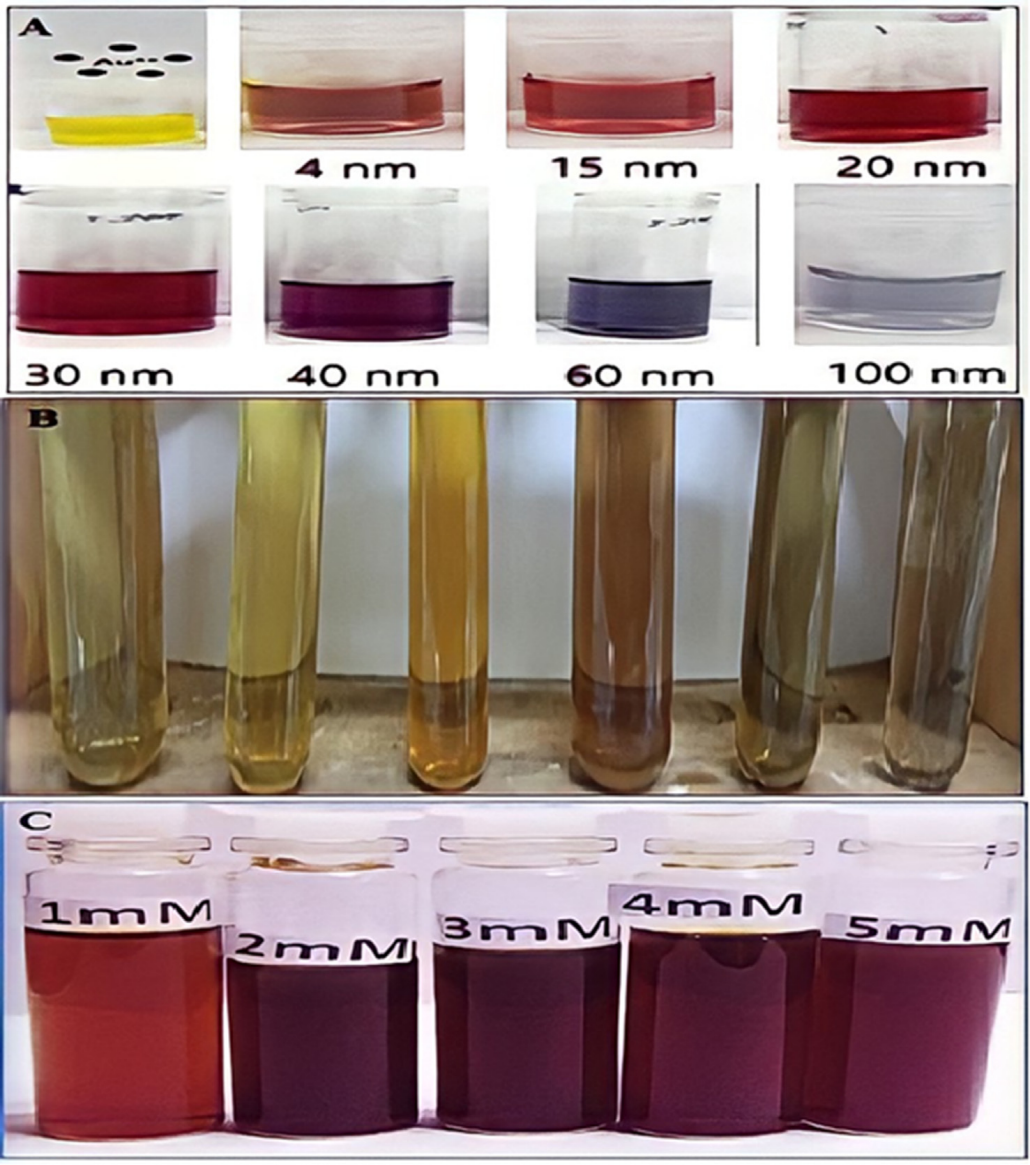
A) Color transformation of AuNPs of different sizes, B) Color of silver nanoparticles, C) *Azadirachta indica* Mediated Biosynthesis of Silver Nanoparticles.

Transition metal complexes has vibrant hues result from electron excitation (Stepanov et al., 2014). Only free electron metals like Au, Ag, Cu, Pt, and alkali metals have visible plasmon resonances that give birth to such bright hues. These plasmon bands are seen in ellipsoids and nanorods (Liz-marz, 2004). Color may be created through atomic electronic transitions. The color transformation determined the synthesis of NPs. SPR is a characteristic of noble metals that gives them distinctive optical features (Ibrahim, 2015). Nanoparticles of gold with a diameter of 5–100 nm (Haiss et al., 2007). While the electrons travel at the same frequency as the light, the plasmon is said to be in resonance, and when they are in resonance, the emitted light is colored (Fig. 8**A**).

The biosynthesized NPs their anticancer action was accomplished in five minutes, and the reduction of gold ions to gold nanoparticles turned the light-yellow solution ruby red, signifying the production of gold nanoparticles (Geetha et al., 2013). The development of a purple tint after combining the plant extract with (HAuCl_4_) solution suggested the creation of Au-NPs (Elia et al., 2014). When the extract was applied, the reaction altered from yellow to violet. The color shift in metallic nanoparticles is caused by the collective oscillation of free conduction electrons (SPR) (Noruzi et al., 2011). The color change indicated the decrease of (HAuCl_4_) and the production of (Au-NPs) due to extract. Color change indicated synthesis of nanoparticles Fig. 8**B** shows a color change caused by the silver nanoparticles' instability (Badi’ah et al., 2019).

**Fig. 9:**
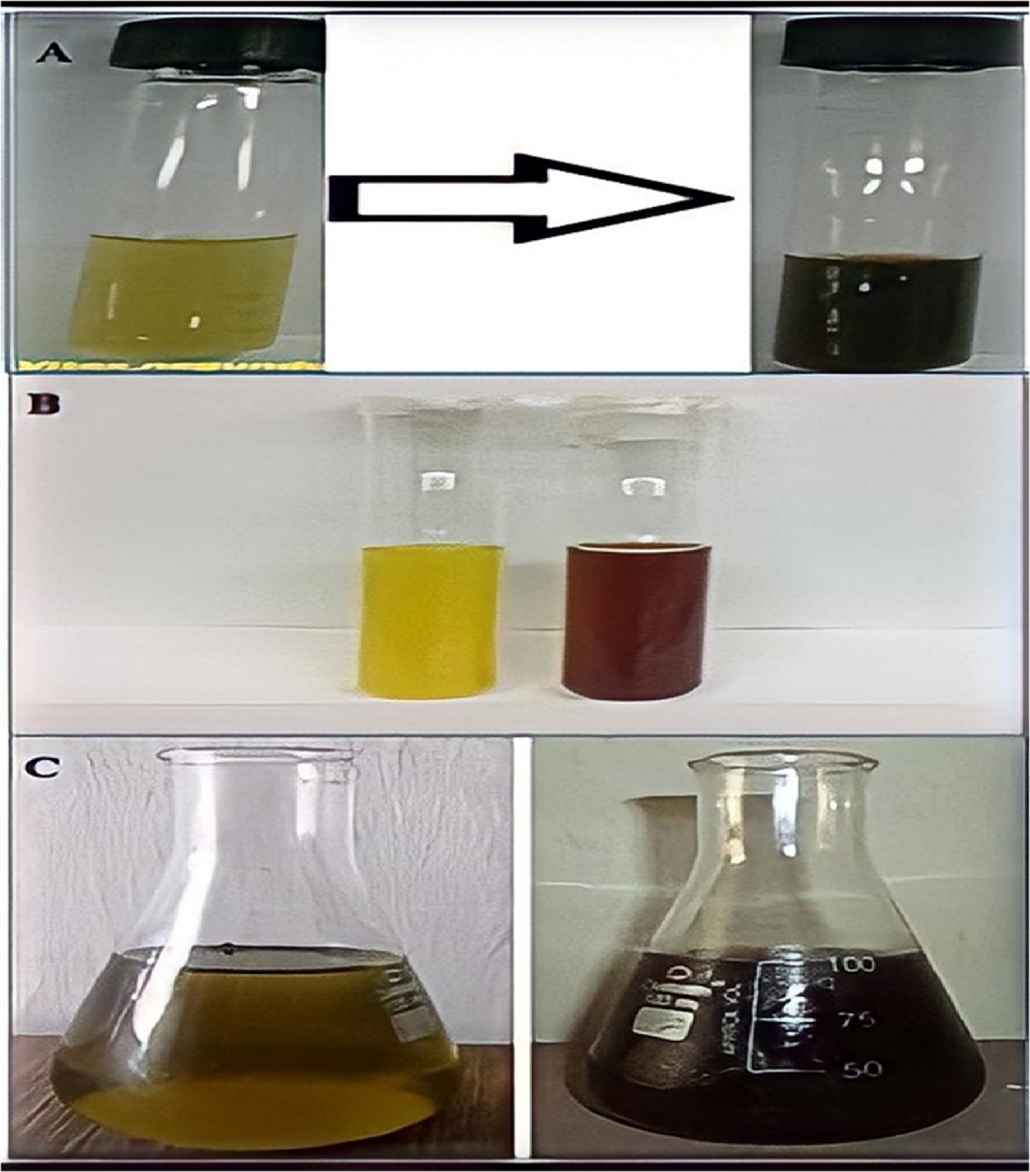
A) Biosynthesis of Turmeric Mediated Ag-NPs B) Vitex Negundo L Mediated Biosynrhesis of Ag-NPs and its Antibacterial Properties C) Green production and antibacterial activity of *Boerhaavia diffusa* Mediated Ag-NPs.

When silver nanoparticles are synthesized from Jatropha curcas seed extract, the surface plasmon absorption bands (SPR) shift from red to reddish yellow with increasing silver nitrate concentration from (103–102) M, and the associated color changes from reddish yellow to reddish brown (Badi’ah et al., 2019, Bar et al., 2009). (Fig. 8**C**) owing to activation of silver nanoparticle surface plasmon vibrations. The brown color was caused by (SPR), a property of silver nanoparticles with absorbance values in the (446–448) nm range (Al-Radadi, 2018). After mixing (AETP) with (AgNO_3_), the mixture became light brown, brown, and eventually brown reddish (Fig. 9**A**). This color shift was accompanied by a change in silver nanoparticle biogenesis (Ag-NPs). The mixture became yellow, then brown, then yellow–brown. Monitoring silver nanoparticle generation using color change and UV–VIS spectroscopy. Fig. 9**B** indicated antibacterial impact of Ag-NPs utilizing Vitex negundo L. The fresh Vitex negundo suspension was yellowish green, but after adding AgNO_3_ and stirring for 48 h at room temperature, the emulsion became dark brown (Zargar et al., 2011). Extract of *B. diffusa* powder (500 mg) was mixed with (1 0 0) ml of dH_2_0 to make an aq extract (Fig. 9**C**). Then, aqueous extract was added to a solution of (90) ml of 0.1 M (AgNO_3_), and the solution turned brown after incubation for 24 h, indicating the formations of silver nanoparticles (Ag-NPs) (Fig. 9**C**) (Kumar et al., 2014).

**Fig. 10:**
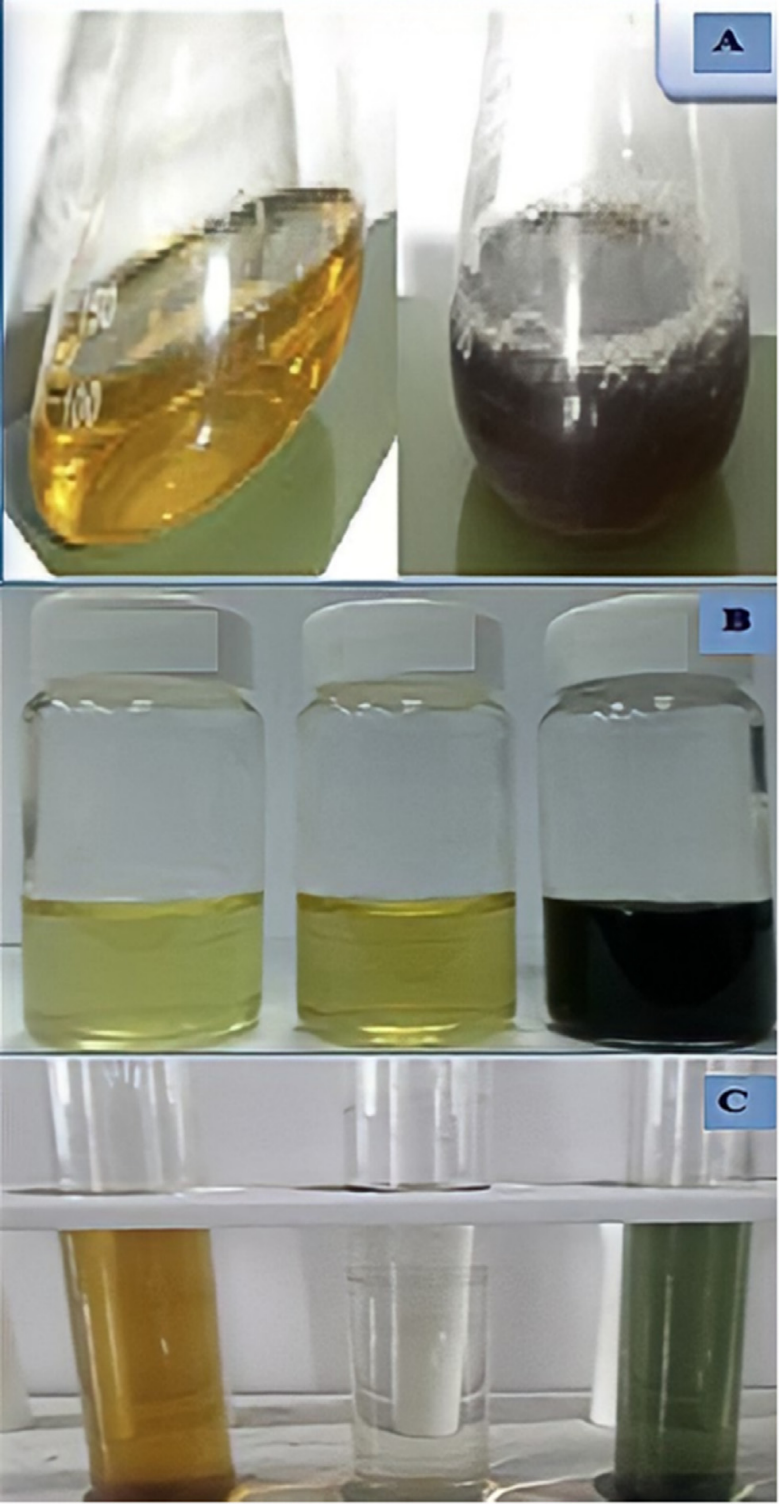
A) Biosynthesis of *Azadirachta indica* Mediated Pt-NPs B) Quercus Glauca mediated Pt-NPs and its electrochemical hydrazine oxidation in water, C) Biosynthesis of *Ocimum sanctum* (Tulsi Mediated Pt-NPs.

Pt-NPs were synthesized using Xanthium stramonium leaf extract in green synthesis and studied biologically at (1 0 0) °C (Thirumurugan et al., 2016). While reducing the reaction temperature, the (Pt^+4^) solution's color changed from yellow to brown (Pt^+2^) and from brown to dark-brown (Pt^0^) (Karthik et al., 2016). Pt nanoparticles were synthesized and characterized using Azadirachta indica leaf extract. The qualitative color shifts from pale yellow to brown show the creation of (Pt-NPs) (Fig. 10**A**).

As illustrated in (Fig. 10**B**), adding Qg extract to (H_2_PtCl_6_·6H_2_O) turned the solution from light yellow to dark brown (Karthik et al., 2016). The electrochemical hydrazine oxidation of Quercus Glauca extract produces green Platinum nanoparticles. The solution transformed from dark green to dark brown with black within minutes of adding Ocimum sanctum Tulsi plant extracts to generate green platinum nanoparticles (Fig. 10**C**). Surface plasmon oscillations in metal nanoparticles induced the optical obliteration of platinum nanoparticles in solution (Prabhu and Gajendran, 2017).

Pt NPs were produced by reducing (H_2_PtCl_6_·6H_2_O) (Al-Radadi, 2019). Green platinum nanoparticles production utilizing Saudi Dates extract and cancer cell therapy.

There are a variety of strategies available for characterizing (MNPs), including the following:

One is the use of an ultraviolet–visible spectroscopy method.

Technique of electron transmission microscopy (TEM).

The X-ray diffractometer (XRD).

FTIR is an infrared imaging technology based on the Fourier transform.

EDX is an X-ray technology that uses energy dispersive radiation.

Using SEM, you can see the smallest details.

HPLC is an acronym for high-performance liquid chromatography.

A method known as zeta potential analysis.

This means that the same wavelength in (TEM), (SEM), and (ZP) Measurements will be obtained for all nanoparticle synthesis to obtain stable nano products. These measurements emphasise the production of nano compounds by fixing four factors and changing one factor. These factors are PH, temperatures, times.

### 2.2 UV–Visible instrument

Optical and electrical characteristics of NPs were assessed by UV (Abdelhalim et al., 2012). UV–Visible spectroscopy may readily demonstrate the creation of (MNPs). The UV–Visible spectroscopy is a critical tool for evaluating metal nanoparticle growth and stability. UV is used to get (SPR) of produced metal. The first batch measured the spectrum for a combination comprising 4 ml of (H_2_PtCl_6_·6H_2_O) solution and varied amounts of extract of Anbara. In the spectra, a clear peak at (380) nm was seen, with a progressive rise in absorbance as the Anbara extract volume rose from 1 to 6 ml.

The researchers discovered a strong peak at (max) (380) nm in the UV absorption spectra of the second batch of Anbara extract and (H_2_PtCl_6_·6H_2_O) solution (0.5–4) ml. As demonstrated in the picture, increasing the concentration of (H_2_PtCl_6_) solution (0.5–4) ml redshifted the peak location. Anbara extract and (H_2_PtCl_6_) were utilized for the first (420) minutes (7 h) before stabilizing the concentration. Ajwa extract and Pt ion were used to measure UV–Visible spectra after the solution was held at room temperature for ten hours. The sharpest peak for UV–Visible spectra was seen at (max) = 321 nm (SPR) with (Pt^0^) using five ml of the aqueous solution (Al-Radadi, 2019).

As the concentration of (HAuCl_4_·3H_2_O) increases, the absorption peak increases and becomes sharper at (max) = (538) nm, which shows the gold nanoparticles UV–Visible spectra using Ajwa extracts of (HAuCl_4_·3H_2_O) (1x10^-2^) M (0.5–1.5) at room temperature after (3) h. At room temperature and after three hours with varying concentrations of Ajwa extract and (1 × 10^-2^) M (HAuCl_4_·3H_2_O), the UV–visible spectra of Au-ANPs with varied concentrations of Ajwa extract are displayed (Al-Radadi & Al-Youbi, 2018b).

During the reduction step, (HAuCl_4_·3H_2_O) ions were easily transformed to Au ions utilizing Licorice root extract. After (150) minutes, the creation of Au nanoparticles worked out effectively, while increasing the amount of Licorice root extract leads in an increase in bright and sharp SPR peak at (549) nm wavelength.

More Au nanoparticles were formed when the (HAuCl_4_·3H_2_O) volume Licorice root extract from (1–4) ml, as shown by a greater absorbance value. The plasmon intensity rose at (540) nm as the amount of (HAuCl_4_·3H_2_O) (3–6) ml increased (Al-Radadi, 2021a).

As the density of silver nitrate (AgNO_3_) rises, the absorption peak becomes sharper and is more apparent at (max) = (454) nm. This was tested by adding different volumes of silver nitrate (1–5) ml to the Cynara scolymus L., extract (5) ml solution (Ag-NPs) formation using constant (AgNO_3_) concentration (5) ml of (0.02) M and various concentrations of extract at room temperature after (3) hours. The absorption peak gets sharper when the density of Cynara scolymus L., extract increases, and a blue shift was observed at (454) nm in the reaction medium, which shows an increase in the mean diameter of (Ag-NPs) (Al-Radadi, 2018).

### 2.3 Transmission electron microscopy (TEM)

For these reasons, transmission electron microscopy (TEM) is one of the best methods for characterizing nanoparticle size, shape, and density (Stepanov et al., 2014). For a resolution of (0.1) nm, it uses an electron beam that passes through the specimen and produces pictures that show the internal structure of the specimen. The light microscope cannot reach this resolution; hence the electron beam is used. TEM contains three key systems:

1. An electron gun and condenser system for focusing the electron beam on the sample.

2. To produce an actual, greatly magnified picture, a series of lenses is used to concentrate electrons travelling through a specimen. This system includes an objective lens, a moveable stage, and objective, intermediate, and projection lenses. (Subramanian et al., 2013).

3. The method for capturing images that transforms an electron picture into something the human eye can see.

**Fig. 11:**
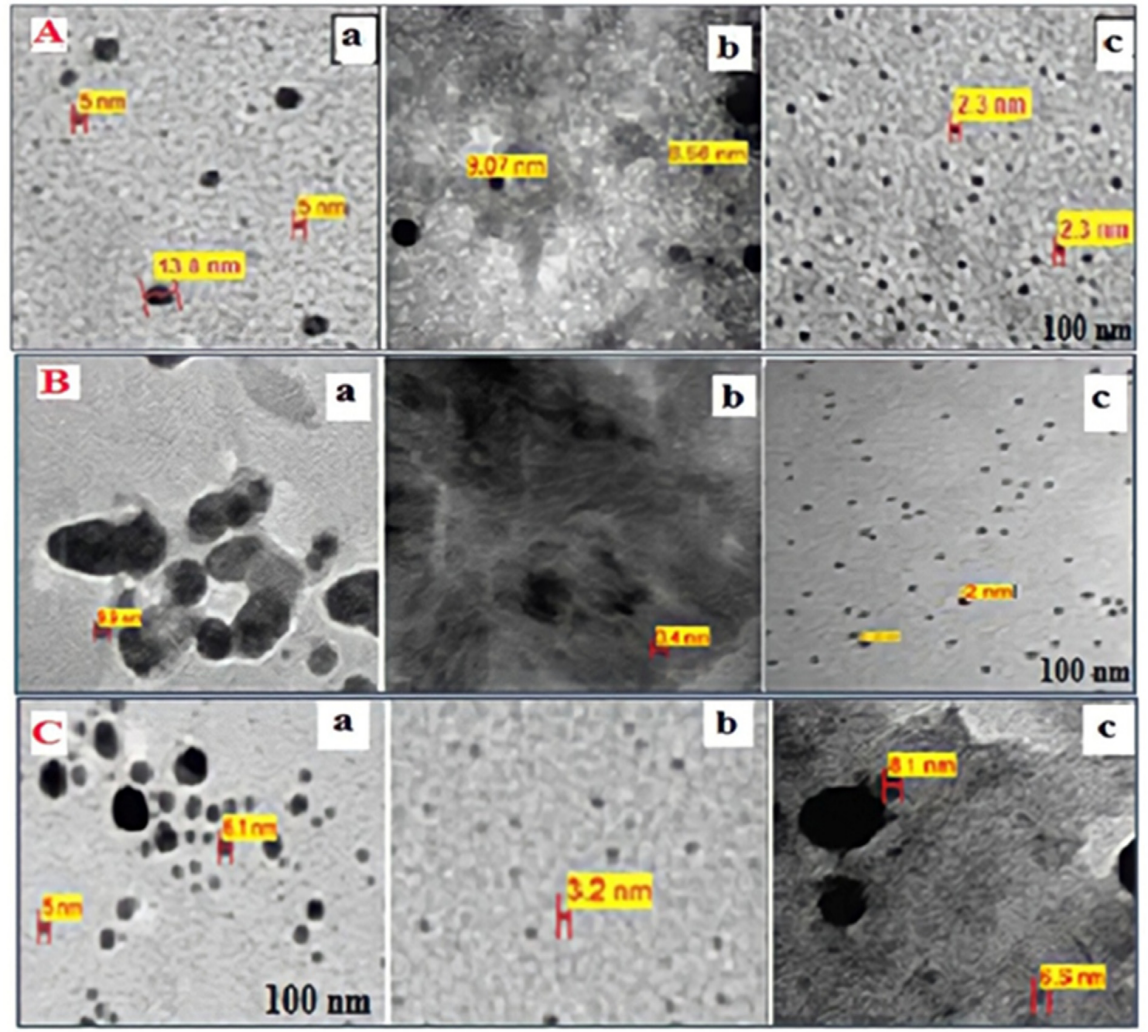
A) TEM micrograph of Pt-NPs at a-4 ml, b-5 ml, and c-6 ml Anbara extract (25) °C, B) Anbara extract (6) ml and (10–3) M (H_2_PtCl_6_·6H_2_O) stock solutions (a) 2 ml (b) 3 ml, and (c) 4 ml, after (7) hours at (25) °C, C) TEM micrograph of (Pt-NPs) (4) ml of (H_2_PtCl_6_) solution and (6) ml of Anbara extract at different time a: (4) hrs, b (7) hrs, c (11) hrs at (25) °C.

It shows some properties of nanoparticles like size, shape, and how it doesn't form agglomerates and how the metal is a capping agent, and the images allow researchers to see samples at the molecular level. The TEM micrograph confirmed the presence of homogeneous H_2_PtCl_6_·6H_2_O (Fig. 11**A**).

TEM images revealed 2.3–3.0 nm in mixes of (6) ml Anbara extract and (2–4) ml (H_2_PtCl_6_·6H_2_O) solutions (Fig. 11**B**) From the (TEM) pictures, it can be seen that the volume of (H2PtCl6) solution added to the Anbara extract lowered the size of the (MNPs), as indicated in (Fig. 11**B**).

For the third batch chemical combination, TEM pictures of reactions were collected at (4), (7), and (11) hours (Fig. 11**C**). The (TEM) pictures of the suggested reaction conditions demonstrate that the intended (Pt-NPs) produced a nearly spherical shape with a particle size of (2.3–3.0) nm (Al-Radadi & Adam, 2020).

For Ajwa, the morphology and size of Au nanoparticles were examined using (TEM) pictures, and the spherical gold nanoparticles had an average diameter of (3.8) nm at the maximum measurement wavelength of (538). (TEM) photographs reveal in the study that these findings have been validated as well as an increase in (HAuCl_4_·3H_2_O) stock solution amounts until it becomes homogenous and the lowest nanoparticle size possible (Al-Radadi & Al-Youbi, 2018b).

TEM images of date extricate blended specimens using (5) and (5) ml of the (AgNO_3_) solution at room temperature after (3) h incubation as shown in (TEM) clearly show that the shape of tested particles is spherical with a formation of narrow size and homogeneous distribution for the spherical Ag nanoparticles as extract concentration is increased (Al-Radadi, 2018).

### 2.4 X-Ray diffraction

**Fig. 12:**
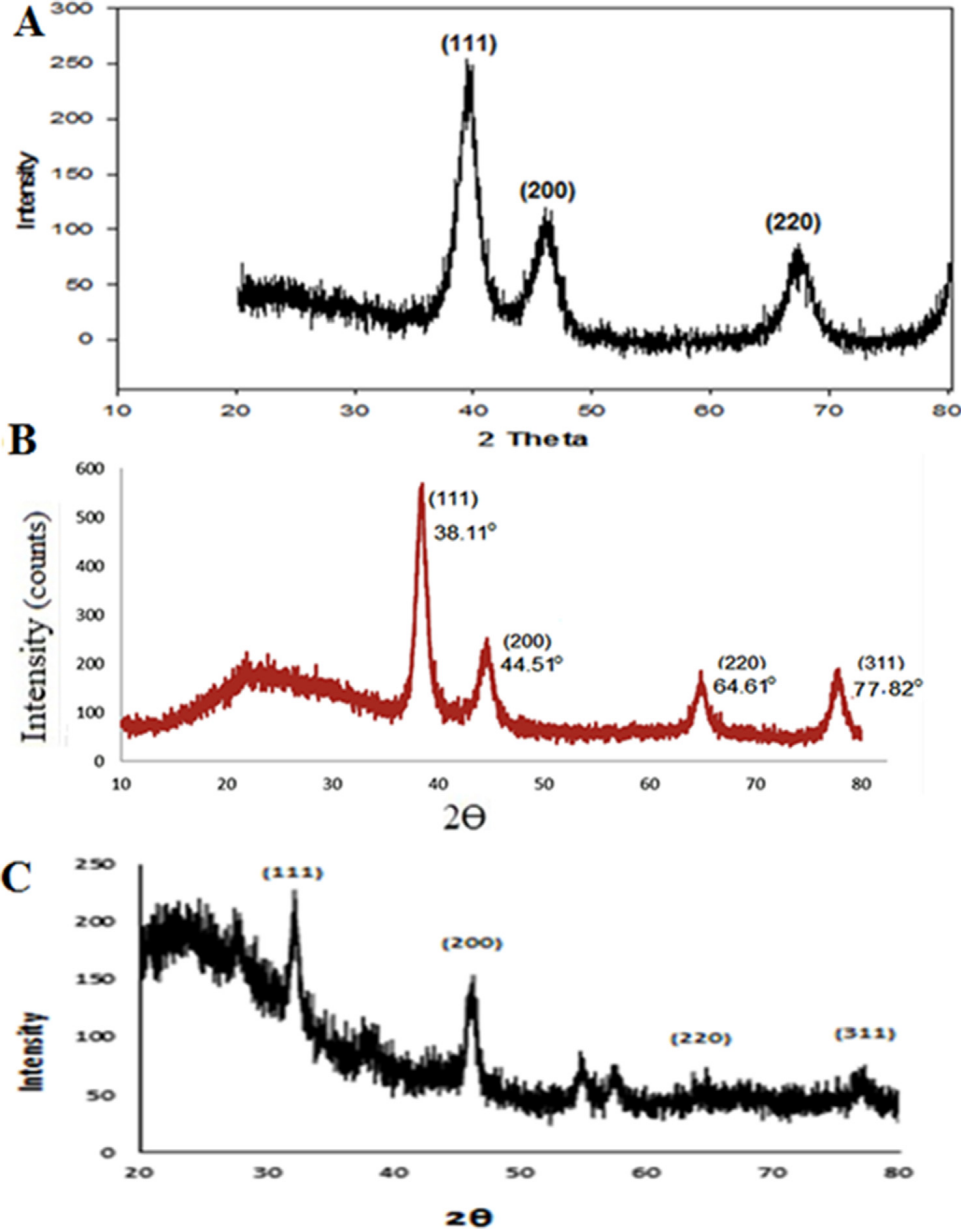
A) X-Ray diffraction pattern of (Pt-NPs) prepared with aqueous Ajwa extract, **B) With Licorice root extract, XRD spectrum of Au-NPs, C) XRay diffraction pattern of Ag-CsL., (NPs).**

XRD is the most significant technique for studying nanomaterials (Bykkam et al., 2015). This technique utilizes X-rays from a cathode-ray tube that are produced with monochromatic radiation, filtered, collimated, and directed at the sample in question in order to perform the diffraction analysis (Bunaciu et al., 2015). It is possible to have access to the morphological and structural information of nanomaterials via X-ray scattering and Bragg diffraction (XRD) can give a high level of precision and accuracy in structural information Because of nanocrystals' size constriction and the existence of intrinsic strain, which results from this confinement, (XRD) examination may validate the crystallinity of the sample by revealing a variety of peaks corresponding to distinct reflection planes (Lavina et al., 2014). The electron micrographs' morphological properties may be linked to the chemical composition using diffracted X-ray intensity measurements**.** For example, in (Fig. 12**A**), the XRD patterns of dried platinum nanoparticles synthesized using Ajwa extract are shown. The (XRD) peaks at two thetas = (39.38°) and (45.88°) correspond to indexed planes (111), (200), and (220), respectively, indicated FCC structure of Pt-NPs. This means the structure of Pt nanoparticles is face centered cubic (As shown by the Scherrer equation, this equation links peak broadening in (XRD) to average particle size, where D represents particle diameter size and k represents a constant equal 1. (0.9) is the X-ray source's wavelength (1.5406 nm); = (2) According to the Debye Scherrer equation, which uses FWHM and diffraction angles corresponding to the lattice plane (111), the average crystallite size is determined to be in the range (1.1–2.5) nm, which is in excellent agreement with TEM images that show a (Pt-NPs) size range of (1.3–2.6) nm (Al-Radadi, 2019; Al-Radadi & Adam, 2020).

It was discovered that by studying the powder (XRD) pattern of gold nano powders, the crystalline nature of gold nano powder could be determined to be (2b = 38.11°, 44.51°, 64.61°, and 77.82°). (Flaxseed@AuNPs) have a face-centered cubic structure, and these indices describe it (Fig. 12**B**). (AuNPs) had a larger average size because they are synthesized in an amorphous environment, but the band associated with (1 1 1) was strong enough to indicate that the synthesized (AuNPs) have crystal nature, and (1 1 1) was the primary orientation (Al-Radadi, 2021a, Al-Radadi, 2021b).

The (XRD) spectrum showed four distinct diffraction peaks at 32.30°, 46.24°, 63.94°, and 76.84°, which correspond to lattice plane values indexed at (1 1 1), (2 0 0), and (2 2 0) and (3 1 1). The average Cynara scolymus L.,@AgNPs crystalline size has been estimated using the well-known Debye Scherrer formula (Fig. 12C). The average crystallite size based on Debye Scherrer equation (Al-Radadi, 2018).

### 2.5 FTIR spectroscopy

**Fig. 13:**
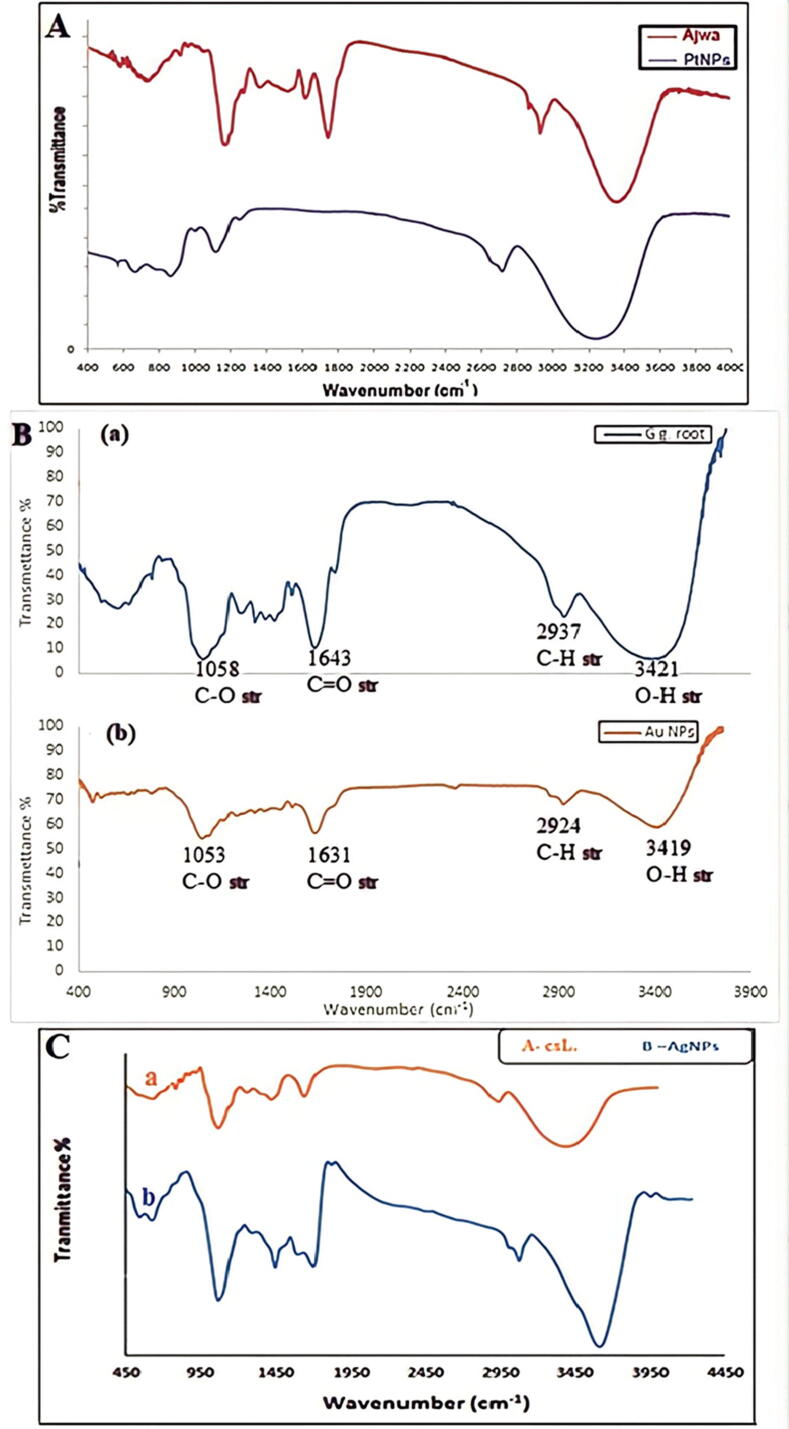
A) FTIR spectra of the dried Ajwa and capped (Pt-NPs) using the Ajwa extract, B) FTIR spectra of (Au-NPs) (b) and Licorice root extract (a), C) FTIR spectra of (A) the dried Cynara scolymus L., and (B) capped (Ag-NPs) using the Cynara scolymus L., extract.

Spectrometers, both classical and modern, give the same information because they both utilize the FT-IR with a Michelson interferometer. The main difference is that modern spectrometers use a Michelson interferometer, which allows all frequencies to reach the detector simultaneously rather than one after the other (Theophanides, 2012). A widely used technique, called FTIR, uses an infrared beam to detect functional groups in materials such as gases, liquids, and solids. FTIR measures the absorption of (IR) radiation by each bond in the molecule and gives a spectrum commonly known as percent transmittance versus wavenumber (cm^−1^) to determine functional groups in a molecule (Sharma et al., 2018). There are different functional groups of polyphenols participating in NPs synthesis as shown in Fig. 13**A**, which shows the (FTIR) spectra of dried Ajwa and capped (Pt-NPs). The (IR) bands observed at (3360), (1760), and (1644) cm^−1^ in dried ajwa is characteristic of the (O-H), (C=O), and (C-N) amide respectively. The stretching modes for (OH), In (Pt-NPs), the disappearance of the strong band at (1760) cm^−1^ indicates that water-soluble polyphenols from Ajwa extract are responsible for the bio reduction and capping of the (Pt-NPs), and the shift in (NH) stretching frequency (2900–2790) cm^−1^ indicates a binding of biomolecules to the (Pt-NPs) through the (NH) group of amino acids in (Pt-NPs) In addition, the band at (1644) cm^−1^ in dried Ajwa assigned as (C-N) amide vibrations is almost gone, indicating the participation of protein amide in the binding to the (Pt-NPs). The appearance of (IR) bands due to (C=O), amide (NH) stretching vibration of the (Pt-NPs) and their shift from that of Ajwa indicate the possibility that (Pt-NPs) are coated with antioxidant molecules in the Ajwa.

In (Fig. 13**B**), the analysis of Licorice root observed many peaks from 3421 to 609) cm^−1^, corresponds to a wide range of vibrations in different functional groups.

Cynara scolymus L. extract and purified (Ag-NPs) have been analyzed by (FTIR) spectroscopy in order to reduce Ag ions by finding and identifying biomolecules in the extract that are responsible; different patterns of vibrations have been discovered and assigned to various functional groups; the extract of Cynara scolymus L. have shown strong expanding vibrations of the (OH) functional group or (NH) stretch vibration amides of protein at (3369.03) cm^−1^ (Fig. 13**C**).

### 2.6 Energy dispersive X-ray (EDX)

Due to its excellent sensitivity in identifying various components in tissues, EDX microanalysis is used in several biological areas of inquiry (Scimeca et al., 2018). A solid's composition may be determined using EDX or EDS by monitoring nuclear emissions, which cause electrons farther away to lose energy and fill the resultant holes. As the vacated lower energy states are filled, each element emits a unique set of (X-Ray) frequencies, which may give qualitative and quantitative information about the near surface m. Several types of photons are produced when the beam interacts with the specimen surface, they vary depending on the surface morphology when coupled with a (EDX) detector, compositional information can be collected (X-Rays), In order to create elemental composition maps of individual samples, EDX detector detects X-ray properties of distinct elements into an energy spectrum (Ellingham et al., 2018). Although other elements were detected, a substantial Pt signal indicated the elemental composition synthesized (Pt-NPs) from Anbara fruits (Al-Radadi & Adam, 2020).

**Fig. 14:**
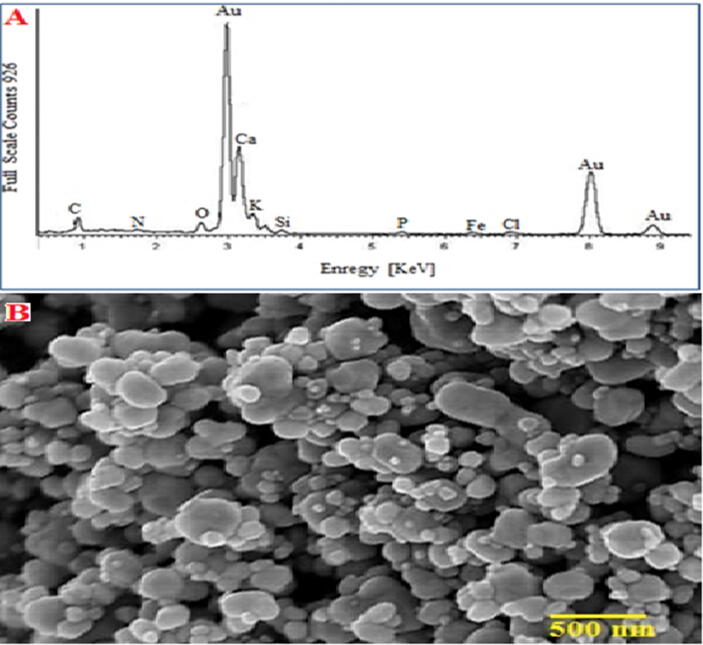
A) EDX analysis of Licorice root@AuNPs, B)SEM Micrograph of Au-NPs.

There were significant optical signals at (3), (8), and (8.9) KeV in (Au-NPs) produced using Licorice root extract, confirming the existence of Au atoms in (EDX) analysis (Fig. 14**A**) Other phytochemicals like oxygen, potassium, and calcium were also found to be present, suggesting that Licorice root compounds like these have a capping function (Al-Radadi, 2021a).

EDX spectra of Ag and Au (NPs) with AL-Madinah Barni fruit is depicted. The spectra revealed strong signals that correspond to the presence of the desired particles, while other peaks were also observed that could be attributed to the presence of extract biomolecules that act as a shell layer for Ag and Au (NPs).

### 2.7 Scanning electron microscopy

An important characterization method is scanning electron microscopy (SEM), which provides spatially resolved data with nanometer scale resolution (Hunnestad et al., 2020). A scale bar is commonly displayed on a (SEM) picture from which the real size of structures in the image may be computed. It can magnify an item between 10 and 300,000 times. In terms of microstructure morphology and chemical composition characterizations, it is one of the most flexible devices accessible (Zhou et al., 2007). It uses electrons instead of photons to take images of objects (Haan et al., 2019). Nanoparticle size, structure, and surface fractures can be determined using scanning electron microscopy (SEM). This type of microscope uses an electron beam to examine the surface of nanoparticles, such as gold nanoparticles (Au-NPs). Fig. 14**B** shows how the prepared (Au-NPs) were sized and surface morphology was imaged using (SEM) analysis.

### 2.8 High performance liquid chromatography (HPLC)

Given its capacity to identify small quantities in materials at semi-micro and trace levels, HPLC has become important analytical methods for analyzing biological systems and medication preparations (Tekkeli & Kiziltas, 2017). To separate, identify, and quantify active chemicals, this particular kind of column chromatography is used in biochemistry and analysis (Sayed, 2021). In recent years, it has become a well-established method utilized in labs all over the world. The development of packing materials used to produce separation has been a major driver of this technique's expansion (Gama et al., 2013). Plant secondary metabolites are phytochemicals or natural antioxidants. Fruits and vegetables contain phenols, which have a particular flavor, taste, and health-promoting properties. Blood pressure-lowering luteolin, rutin, and kampherol Glycosides In addition, linamarin, pinoresinol, and lorcinadols include the most glycosides, and organic acids such as succinic, ascorbic, gallic, and fumaric acids contribute to the acidity of the Licorice root.

The sterols contents of Glycyrrhiza glabra tincture and mass fragmentation are illustrated (Al-Radadi, 2021a). The tincture includes three studied sterols: beta-sitosterol, dihydrostig-masterol, and ergosterol. The licorice root plant contained 17 amino acids out of 23, with substantial changes in their amino acid concentrations.

The mineral composition of licorice root varies with variation Calcium (387.1 mg/100 g) was discovered to be the most prevalent mineral in Licorice root, followed by Potassium, Silicone and Phosphorus. The root had iron, sulfur, and magnesium. The mineral components A (GC–MS) analysis on Licorice root was undertaken to determine its aromatic components. The root contains 28 distinct chemicals, the most prevalent being E, E, and Z-1,3,12-Nonadecatriene-5. The root's carbohydrate sources include E, E, and Z-1,3,12-Nonadecatriene-5 (Al-Radadi, 2021a).

**Fig. 15:**
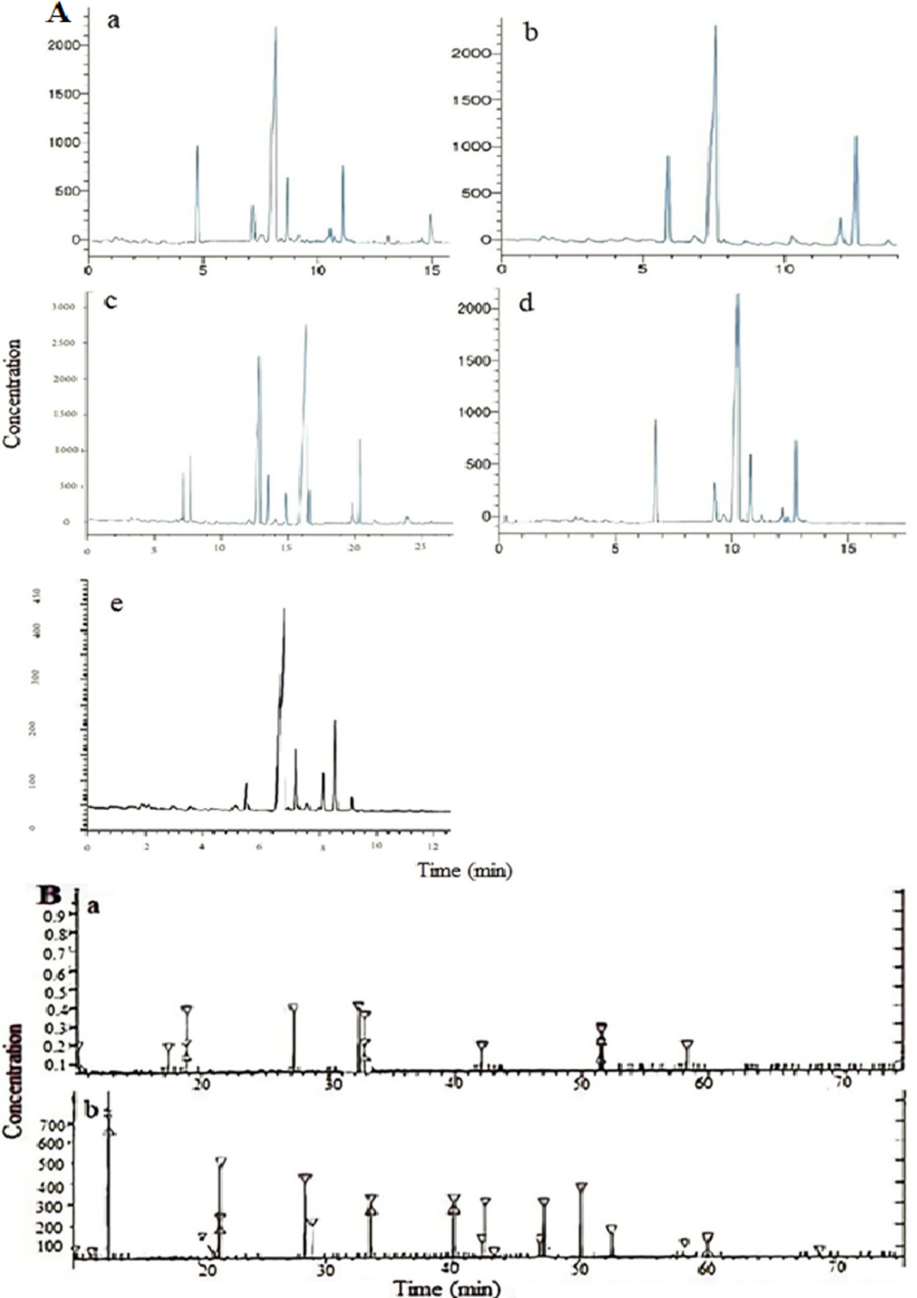
A) HPLC chromatograms of Phenolics (a), flavonoids (b), carotenoids (c), Organic acids (d), glycosides (e) in flaxseed, B) HPLC charts for a) non-essential and b) essential amino acid separation in Flaxseed extract.

**Fig. 16:**
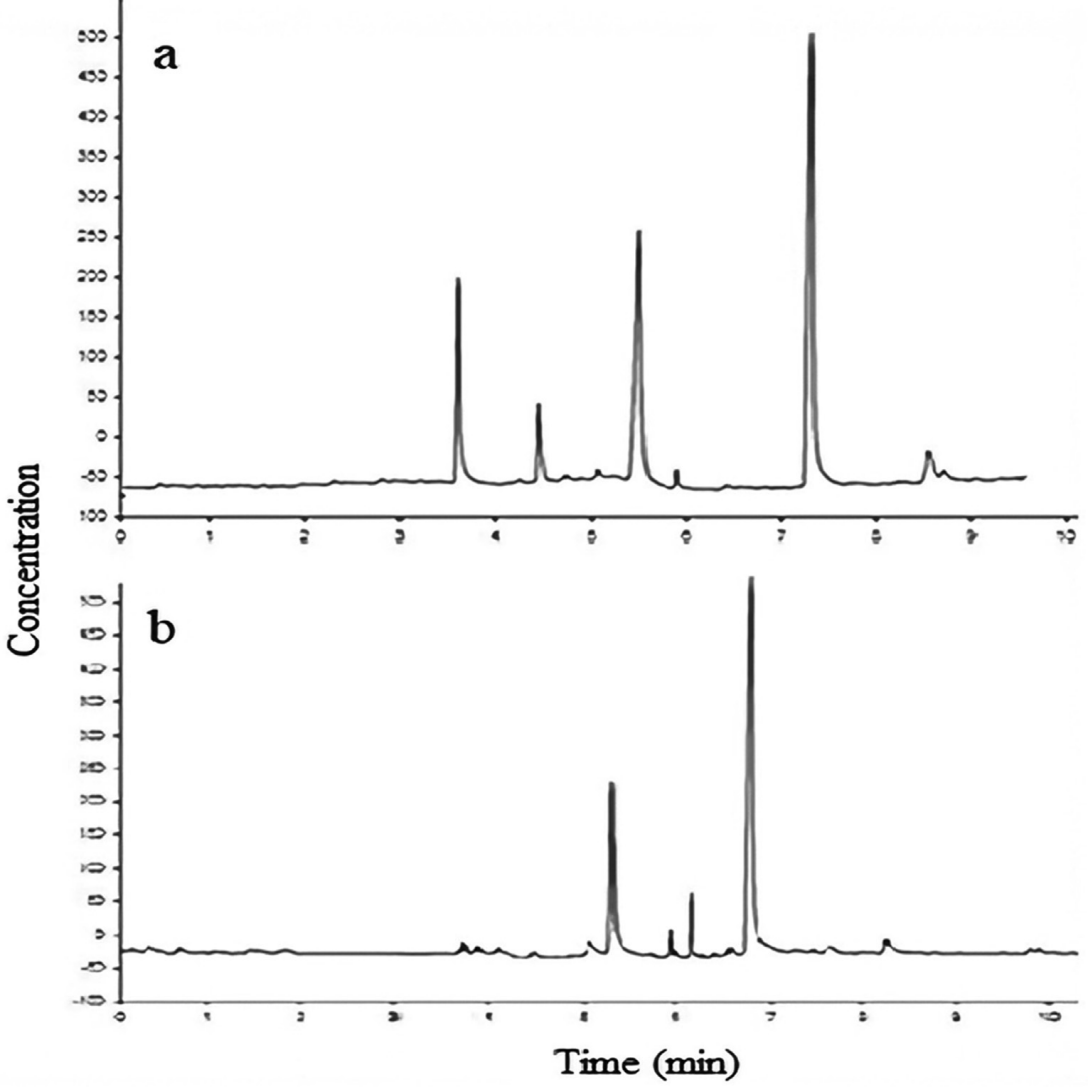
HPLC chromatograms of water soluble vitamins (a) fat soluble vitamins (b) of flaxseed.

**Fig. 17:**
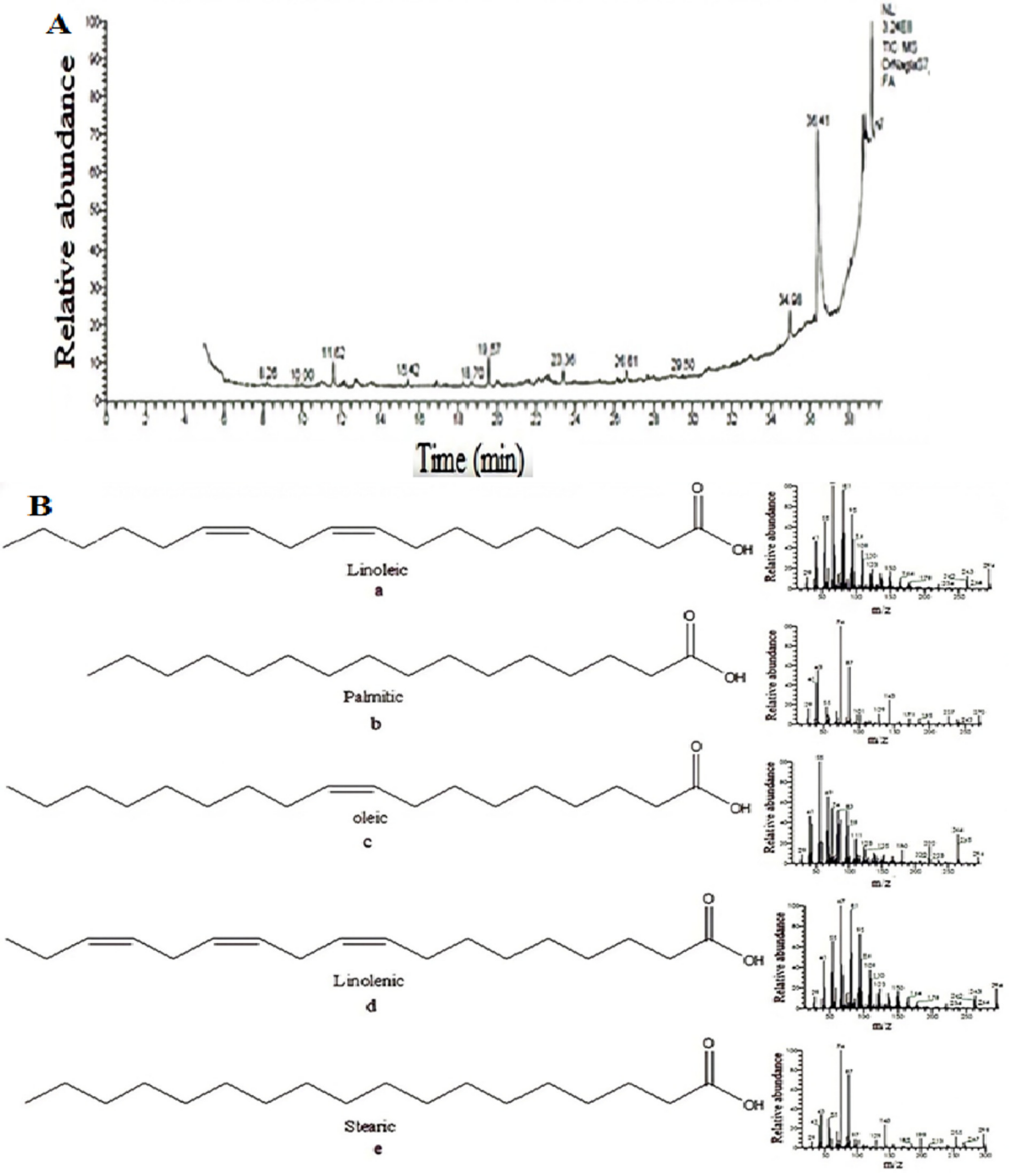
(A) Chart for Separation of fatty acids in flaxseed and (B) Mass Fragmentation of the separated of Linoleic (a), Palmitic (b), Oleic (c), Linolenic (d), Stearic (e).

Sugar content of Ajwa dates from Al-Madinah area. Dates are clearly a sugary food item due to their high sugar content. Anbar and Barni had the most glucose (312.908 and 57.3%), whereas Al-Madinah dates had the least fructose and sucrose (Al-Radadi, 2019; Al-Radadi and Adam, 2020, Al-Radadi and Al-Youbi, 2018a). In other words, all minerals were present, although in different amounts in the studied cultivars. Barni had a high mineral concentration. Because of its nutritional value and bioactive components, flaxseed composition is reported by HPLC. The flaxseed content varies with the growth climate and seed processing method. Flaxseed contains phenolics such flavonoids and phenolic and amino acids, as well as lignans (SDG) in Fig. 15**A, B**, which are abundant in flaxseed. Flaxseed includes antioxidants, amino acids, carotenoids, organic acids, minerals, and vitamins (Fig. 16), in addition to a high concentration of fatty acids in its seed oil (Fig. 17**A, B)**. Flaxseed contains coumaric acid, syringe Nic acid, sinapic acid, and ferulic acid. Caffeic acid was found to be the most prevalent phenolic component in flaxseed. More specifically, large amounts of linamarin (37.12) mg/g and pinanediol (24.55) mg/(100)g were isolated and described, whereas low concentrations of lorcinadol (8.25) mg/g and threonine (5.13) mg/(100)g were identified. In the Flaxseed depicted, seven fatty acids were discovered, including linoleic and palmitic acids, oleic and linolenic acids, stearic and pentadecanoic and heptadecanoic acids (Al-Radadi, 2021b).

### 2.9 Zeta potential (ZP)

**Fig. 18:**
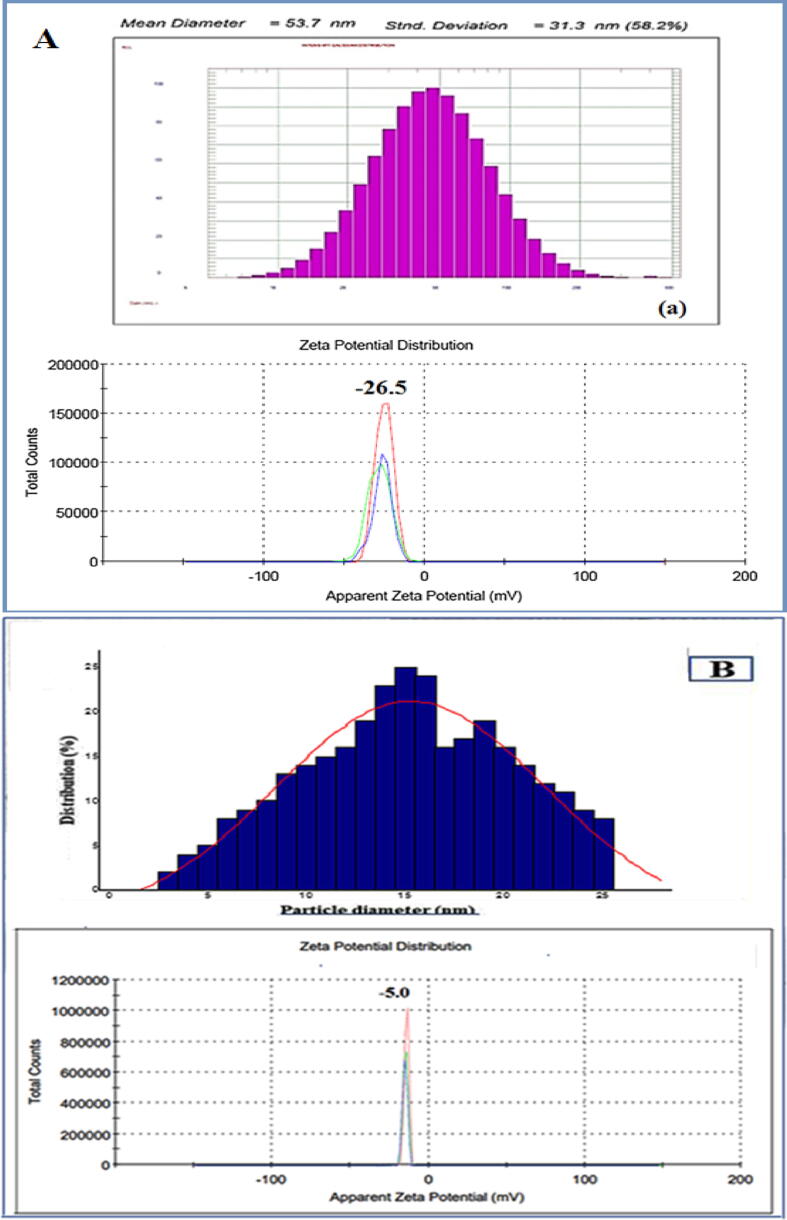
(A) DLS image (a) and (ZP) (b) of (Au-NPs) synthesized by Licorice root extract, B) corresponding size distribution graph (A) and (ZP) (B) of (Fs-Au-NPs).

Zeta potential, the electrostatic potential at a particle's surface, is measured by looking at how fast particles travel in a dc electric field (Favela-Camacho et al., 2019). ZP is a widely used characterization method for nanometer-sized liquid objects such as drugs, inks, and foams. Stabilized particles have a zeta potential of (30) mV. (Vogel et al., 2017). ZP is the shear plane electrostatic potential. The physical stability of colloidal dispersions (ZP) was investigated using a zeta sizer nano (ZS). In this investigation (n = 3), samples were diluted 100 times in ultrapure water before being examined. Each test has 30 iterations (Pereira et al., 2018). DLS is used for nanoparticle hydrodynamic size and surface charge (Bhattacharjee, 2016). The average (ZP) was obtained (-26.5), clearly suggesting that the (Au-NPs) were stable at (25 °C) (Fig. 18**A**) (Al-Radadi, 2021a). They were made from licorice root extract having size of (53.7) nm.

(AuNPs) were synthesized from flax seed using a green biosynthesis method, which revealed that the nanoparticles' sizes were confirmed by a histogram investigation (Fig. 18**B**) and that the average (ZP) of three replicate analyses showed that the nanoparticles (AuNPs) were stable at (25 °C). Previous studies have found similar stability results for (AuNPs), which are in agreement with the current findings (Al-Radadi, 2021b).

## 3 Conclusion

This study topic is more popular in the recent decade because of the high efficiency of plant extracts, as well as the ease, economy, speed, and eco-friendly green synthetic approach of NPs, because of its simplicity, ease of use, low cost, easy scalability, and use of harmlessness, green synthesis was found to be a viable alternative for synthesizing from plants in this project. This is confirmed by the mention of certain research in the project. like the green synthesis of (Au-NPs) with Licorice root, (Ag-NPs) with Artichoke, (Au-NPs) with Flaxseed, (Au-NPs) with Ajwa, (Pt-NPs) with Saudi Dates, (Pt-NPs) with Anbara, and (Ag-NPs), (Au-NPs) with AL-Madinah Barni, by characterizing it.

There are several applications such as UV, XRD, SEM, TEM, EDX, FTIR, DLS, and HPLC by which nanoparticles synthesis can be confirmed.

## Declaration of Competing Interest

The authors declare that they have no known competing financial interests or personal relationships that could have appeared to influence the work reported in this paper.
